# Interception of host fatty acid metabolism by mycobacteria under hypoxia to suppress anti-TB immunity

**DOI:** 10.1038/s41421-021-00301-1

**Published:** 2021-10-05

**Authors:** Hua Yang, Fei Wang, Xinya Guo, Feng Liu, Zhonghua Liu, Xiangyang Wu, Mengmeng Zhao, Mingtong Ma, Haipeng Liu, Lianhua Qin, Lin Wang, Tianqi Tang, Wei Sha, Yang Wang, Jianxia Chen, Xiaochen Huang, Jie Wang, Cheng Peng, Ruijuan Zheng, Fen Tang, Lu Zhang, Chunyan Wu, Stefan H. Oehlers, Zhigang Song, Jialei She, Hua Feng, Xunwei Xie, Baoxue Ge

**Affiliations:** 1 Shanghai Key Laboratory of Tuberculosis, Shanghai Pulmonary Hospital, Tongji University School of Medicine, Shanghai, China; 2 Department of Microbiology and Immunology, Tongji University School of Medicine, Shanghai, China; 3 Tuberculosis Center for Diagnosis and Treatment, Shanghai Pulmonary Hospital, Tongji University School of Medicine, Shanghai, China; 4 State Key Laboratory of Genetic Engineering, Institute of Genetics, School of Life Science, Fudan University, Shanghai, China; 5 Department of Pathology, Shanghai Pulmonary Hospital, Tongji University School of Medicine, Shanghai, China; 6 Tuberculosis Research Program at the Centenary Institute, The University of Sydney, Sydney, NSW Australia; 7 Eastern China Center for Pathogen Discovery and Research, Shanghai Public Health Clinical Center, Fudan University, Shanghai, China; 8 Omics Core of Bio-Med Big Data Center, CAS-MPG Partner Institute for Computational Biology, Shanghai Institutes for Biological Sciences, Chinese Academy of Sciences, Shanghai, China; 9 China Zebrafish Resource Center, Institute of Hydrobiology, Chinese Academy of Sciences, Wuhan, Hubei China

**Keywords:** Innate immunity, Epigenetics

## Abstract

Pathogenic mycobacteria induce the formation of hypoxic granulomas during latent tuberculosis (TB) infection, in which the immune system contains, but fails to eliminate the mycobacteria. Fatty acid metabolism-related genes are relatively overrepresented in the mycobacterial genome and mycobacteria favor host-derived fatty acids as nutrient sources. However, whether and how mycobacteria modulate host fatty acid metabolism to drive granuloma progression remains unknown. Here, we report that mycobacteria under hypoxia markedly secrete the protein Rv0859/MMAR_4677 (Fatty-acid degradation A, FadA), which is also enriched in tuberculous granulomas. FadA acts as an acetyltransferase that converts host acetyl-CoA to acetoacetyl-CoA. The reduced acetyl-CoA level suppresses H3K9Ac-mediated expression of the host proinflammatory cytokine *Il6*, thus promoting granuloma progression. Moreover, supplementation of acetate increases the level of acetyl-CoA and inhibits the formation of granulomas. Our findings suggest an unexpected mechanism of a hypoxia-induced mycobacterial protein suppressing host immunity via modulation of host fatty acid metabolism and raise the possibility of a novel therapeutic strategy for TB infection.

## Introduction

Tuberculosis (TB) caused by *Mycobacterium tuberculosis* (*M. tuberculosis*) is associated with 10 million active cases and 1.4 million deaths annually^1^. One hallmark of TB is the formation of caseous necrotic granulomas^2^, which are organized aggregates of macrophages and other immune cells that serve as niches for the bacteria to obtain nutrients or evade anti-TB immunity, and provide a source of mycobacteria for later reactivation and dissemination^3,4^.

Low oxygen tension is strongly correlated with the formation of hard, fibrous, and hypoxic granulomas^5^. Mycobacteria must adapt to hypoxia in order to survive within granulomas^6–8^. Hypoxia induces widespread transcriptional changes of mycobacterial genes that are associated with a metabolically altered state and cause the bacteria to enter into a non-replicating “quiescent” state that is tolerant of antibiotic treatment^7,9^. Mycobacteria utilize the ESX-1 type VII and SecA secretion system to transport effector proteins across their cell wall into host immune cells^10^. However, whether and how the secreted mycobacterial proteins are induced under hypoxia to promote the adaption to the hostile environment remains unknown.

The expression profiles of mycobacterial and host genes have been well characterized during granuloma progression^11^. The zebrafish-*Mycobacterium marinum* (*M. marinum*) platform has been used to study the role of mycobacterial components including early secretory antigenic target-6 (ESAT-6), trehalose 6,6’-dimycolate (TDM), and host factors including matrix metalloprotease 9 (MMP9), tumor necrosis factor-alpha (TNFα), and vascular endothelial growth factor (VEGF) in the formation and progression of granulomas^12–17^. However, the signaling mechanisms underlying the interaction of hypoxia-induced mycobacterial secreted proteins with host metabolic factors in the regulation of anti-TB immunity during granuloma progression remain unexplored.

In this work, we profile the hypoxia-induced secreted proteins of *M. tuberculosis* and examine their roles in the progression of granuloma. We show that hypoxia strongly induces the secretion of the mycobacterial Rv0859 (Fatty-acid degradation A, FadA) protein, which reduces the level of host acetyl coenzyme A (acetyl-CoA) to suppress histone acetylation-mediated production of proinflammatory cytokines. This in turn promotes the survival of mycobacteria in granuloma for persistent infection.

## Results

### Mycobacterial FadA is induced by hypoxia

**Fig. 1:**
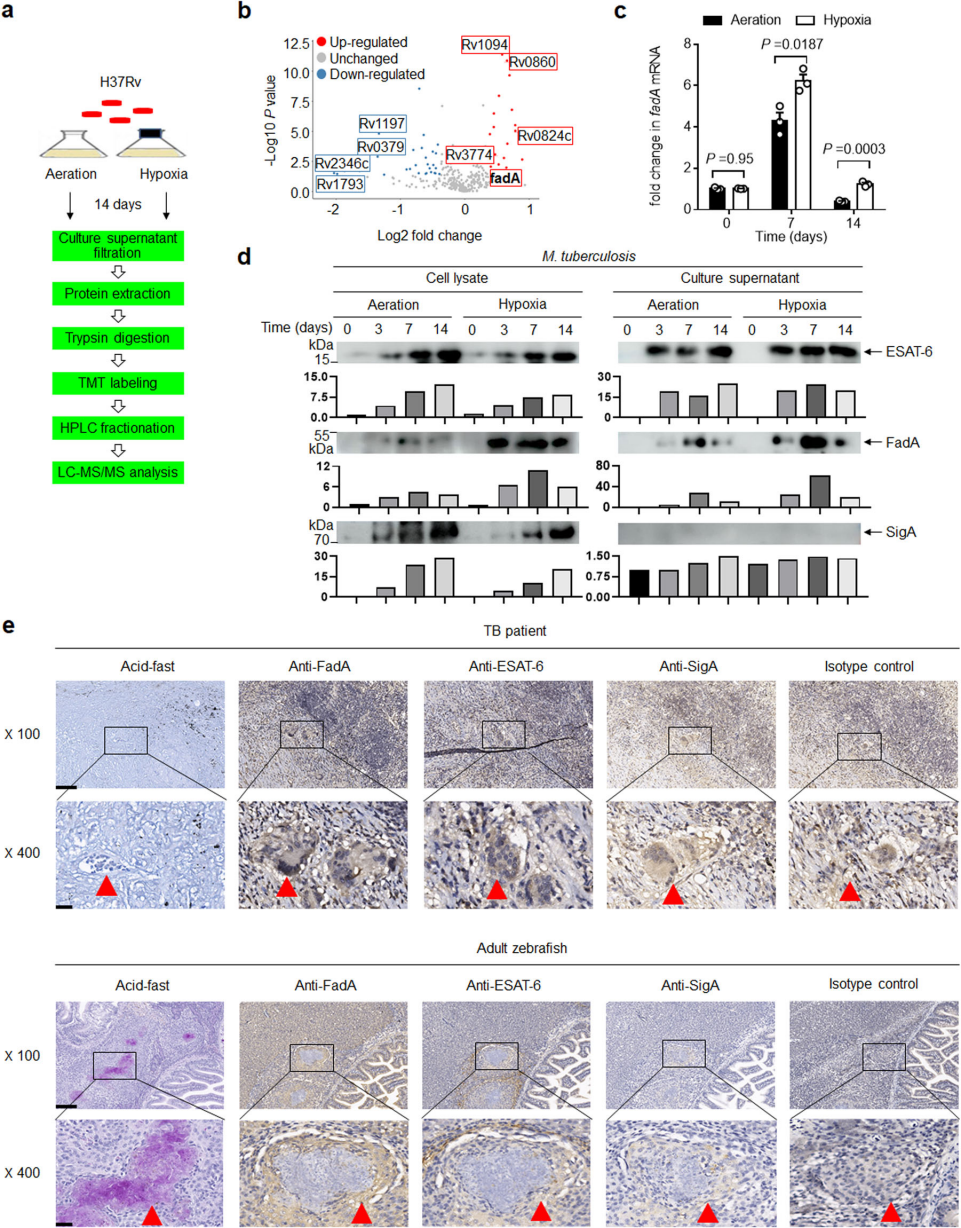
Hypoxia induces FadA. **a** The pathway of quantitative proteomic analysis to examine alterations in the protein profile of H37Rv culture filtrate using the Wayne model of aeration or hypoxia in vitro. **b** Volcano map of identified secreted proteins based on the fold change (Log2) and *t* test *P*-value (−Log10) by comparing hypoxia with aeration. **c** qPCR analysis of Rv0859 (*fadA*) mRNA from H37Rv incubated under aeration and hypoxia for 0, 7, or 14 days in vitro (mean ± SEM). Data are representative of one experiment with at least three independent biological replicates; each circle represents one technical repeat. Bar charts show means. **d** Immunoblot (IB) of culture filtrate and cell lysate from H37Rv incubated under aeration and hypoxia for 0, 3, 7, or 14 days with anti-FadA, anti-ESAT-6, or anti-SigA antibodies at a 1:1000 dilution. Bars represent densitometric analysis of band intensity. **e** Immunolocalization of FadA in lung granuloma sections from TB patients and whole fish sections of *M. marinum* infected adult zebrafish with anti-FadA polyclonal antibody at a 1:100 dilution and anti-rabbit secondary antibody labeled with HRP at a 1:200 dilution (scale bar, 100 μm (top) and 20 μm (bottom)), compared with anti-ESAT-6 polyclonal antibody at a 1:200 dilution, isotype polyclonal control antibody at a 1:100 dilution, anti-SigA antibody labeled with HRP at 1:100 dilution and acid-fast staining (scale bar, 100 μm (top) and 20 μm (bottom)). The red triangle indicates multinucleated giant cells. Results of **d**, **e** are representative of three independent experiments. Two-tailed unpaired Student’s *t* test (**c**) was used for statistical analysis.

To investigate whether hypoxia specifically induces the secretion of mycobacterial proteins, we performed quantitative proteomics analysis (Fig. 1a) of *M. tuberculosis* strain H37Rv culture filtrate following the Wayne and Hayes model in vitro^18,19^. Hypoxia increased the production of 22 secreted proteins (Supplementary Table S1) and inhibited the production of 29 secreted proteins (Supplementary Table S2; Fig. 1b). Kyoto Encyclopedia of Genes and Genomes (KEGG) metabolic pathway analysis showed that upregulated proteins under hypoxia were particularly associated with fatty acid metabolism (Supplementary Fig. S1a). Since fatty acid metabolism-related genes are relatively “overrepresented” in the *M. tuberculosis* genome, and fatty acid metabolism plays an important role in *M. tuberculosis* infection^20,21^, we then focused on the five fatty acid metabolism-related genes Rv0824c, FadA, Rv0860, Rv1094, and Rv3774 for further study. The mRNA levels of these five genes were evaluated, with the expression of FadA being most significantly increased by hypoxia at 7 or 14 days of in vitro culture (Fig. 1c; Supplementary Fig. S1b–e). Consistent with this, the production and secretion of FadA protein in H37Rv or *M. marinum* Aronson (BAA-535) were also significantly induced by hypoxia, as determined by our custom-made anti-FadA-specific polyclonal antibody, positive control anti-ESAT-6 antibody, and negative control anti-SigA antibody (Fig. 1d; Supplementary Fig. S1f). Caseous necrotic granuloma is a hallmark structure of TB that provides a hypoxic environment for the persistent infection of *M. tuberculosis*^4,7,9,22–24^. Infection of zebrafish with *M. marinum* provides a well-established model of hypoxic and necrotic tuberculous granuloma^23^. Immunohistochemical staining of granulomas from TB patients and *M. marinum*-infected adult zebrafish revealed that FadA was highly enriched in the multinucleated giant cells around the center of solid granulomas or caseous granulomas, but not detected in the pathological sections of granulomas from lung cancer patients or whole fish sections of uninfected adult zebrafish, in contrast to the findings for ESAT-6, SigA and isotype controls (Fig. 1e**;** Supplementary Fig. S1g, h). Together, these results demonstrate that FadA is a hypoxia-induced *M. tuberculosis* protein.

### FadA enhances granuloma necrosis

**Fig. 2:**
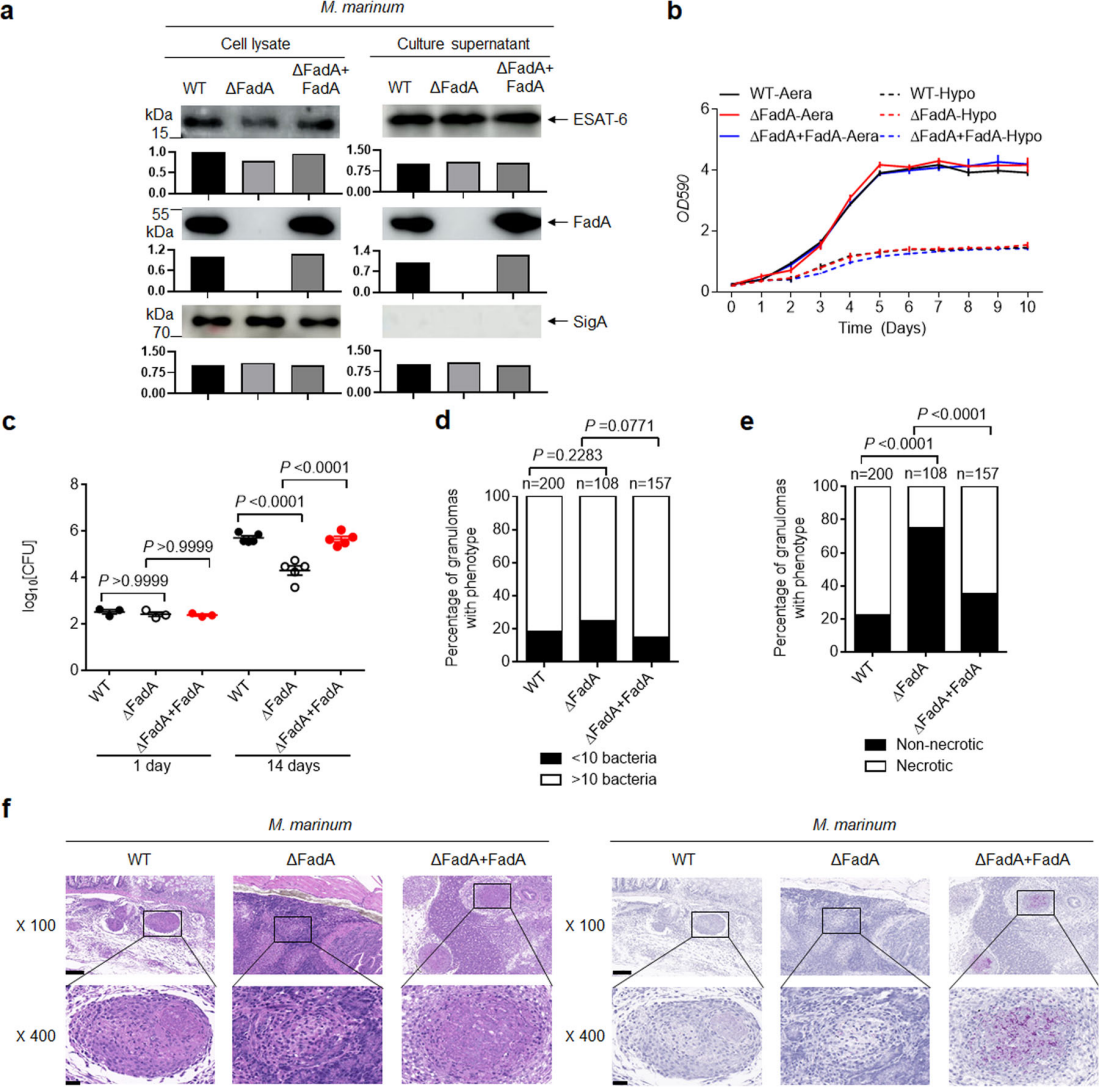
FadA enhances the survival of mycobacteria in granulomas. **a** IB of cell lysate and culture filtrate of WT, ΔFadA, or ΔFadA + FadA *M. marinum* strains with anti-FadA, anti-ESAT-6, and anti-SigA antibodies at a 1:1000 dilution. Bars represent densitometric analysis of band intensity. Results are representative of three independent experiments. **b** Growth curve for in vitro growth of WT, ΔFadA, or ΔFadA + FadA *M. marinum* strains under aeration or hypoxia at 30 °C for 10 days. **c**–**f** Adult zebrafish were intraperitoneally infected with roughly 200 CFU per fish of WT, ΔFadA, or ΔFadA + FadA *M. marinum* strains for 1 or 14 days. Histopathology was assessed in the whole fish sections with bacterial titers (**c**; mean ± SEM of *n* = 3 fish infected for 1 day or *n* = 5 fish infected for 14 days), comparison of granulomas between WT, ΔFadA, or ΔFadA + FadA *M. marinum*-infected adult zebrafish scored for *M. marinum* burden as less than 10 or 10 or more bacteria (**d**), or percentage of necrotic granulomas in each fish (**e**) and H&E or acid-fast staining from zebrafish infected for 14 days (**f**; scale bar, 100 μm (top) and 20 μm (bottom)). “*n*” in **d**, **e** was the total number of granulomas for each strain infected fish. A total number of zebrafish analyzed: five (WT), five (ΔFadA), five (ΔFadA + FadA). Data in **b**–**f** represent one experiment with at least three independent replicates. One-way ANOVA with Bonferroni’s multiple comparisons test (**c**) and Fisher’s exact test (**d**, **e**) was used for statistical analysis.

Considering the high abundance of FadA in granulomas and its specific induction under hypoxia, we next examined the functional relevance of FadA during the formation of tuberculous granulomas. We deleted FadA from *M. marinum* to generate a mutant *M. marinum* (ΔFadA), and complemented *M. marinum* (ΔFadA) with FadA to generate a FadA complemented strain (ΔFadA + FadA) (Fig. 2a), whose growth rate under either aeration or hypoxia showed no difference from that of wild-type (WT) *M. marinum* (Fig. 2b). In accordance with previously described methods^15^, acid fast staining of granulomas was carried out to estimate the bacterial burden in granulomas, and hematoxylin and eosin (H&E) staining was used to score granulomas for the presence of necrotic regions. As reported previously^2,3^, necrotic granulomas show a greater tendency to progress than solid ones. Granulomas of different *M. marinum*-infected adult zebrafish scored for *M. marinum* burden as less than 10 or 10 or more bacteria and the percentage of necrotic granulomas were quantified and compared based on the staining results. The total number of granulomas for each strain was counted and shown with “*n*”, and the different number of granulomas between different groups also reflected the pathology of *M. marinum*-infected zebrafish. Adult zebrafish infected with *M. marinum* (ΔFadA) had a much lower bacterial burden at 14 days post infection and an increased fraction of low-burden or non-necrotic granulomas than WT *M. marinum* or *M. marinum* (ΔFadA + FadA) strains (Fig. 2c–f).

It has also been shown that C3HeB/FeJ mice infected with *M. tuberculosis* develop well-circumscribed TB lung granulomas with central necrosis and tissue hypoxia^25^. We generated a FadA deletion mutant of *M. tuberculosis* strain H37RvΔFadA and a FadA complemented strain H37Rv(ΔFadA+FadA) (Supplementary Fig. S2a), whose growth rate also showed no difference from that of H37Rv (Supplementary Fig. S2b). The lung tissues from C3HeB/FeJ mice 4 weeks post infection with the H37RvΔFadA strain had a much lower bacterial burden (the difference in log_10_ means between H37Rv and H37RvΔFadA was −1.575 ± 0.04916; the difference in log_10_ means between H37RvΔFadA and H37Rv(ΔFadA + FadA) was −1.147 ± 0.04838) and far fewer pathological lesions than those infected with the WT H37Rv or H37Rv(ΔFadA + FadA) strain (Supplementary Fig. S3). Together, these results demonstrate that FadA enhances the survival of mycobacteria in infected zebrafish or mice.

### FadA inhibits IL-6

**Fig. 3:**
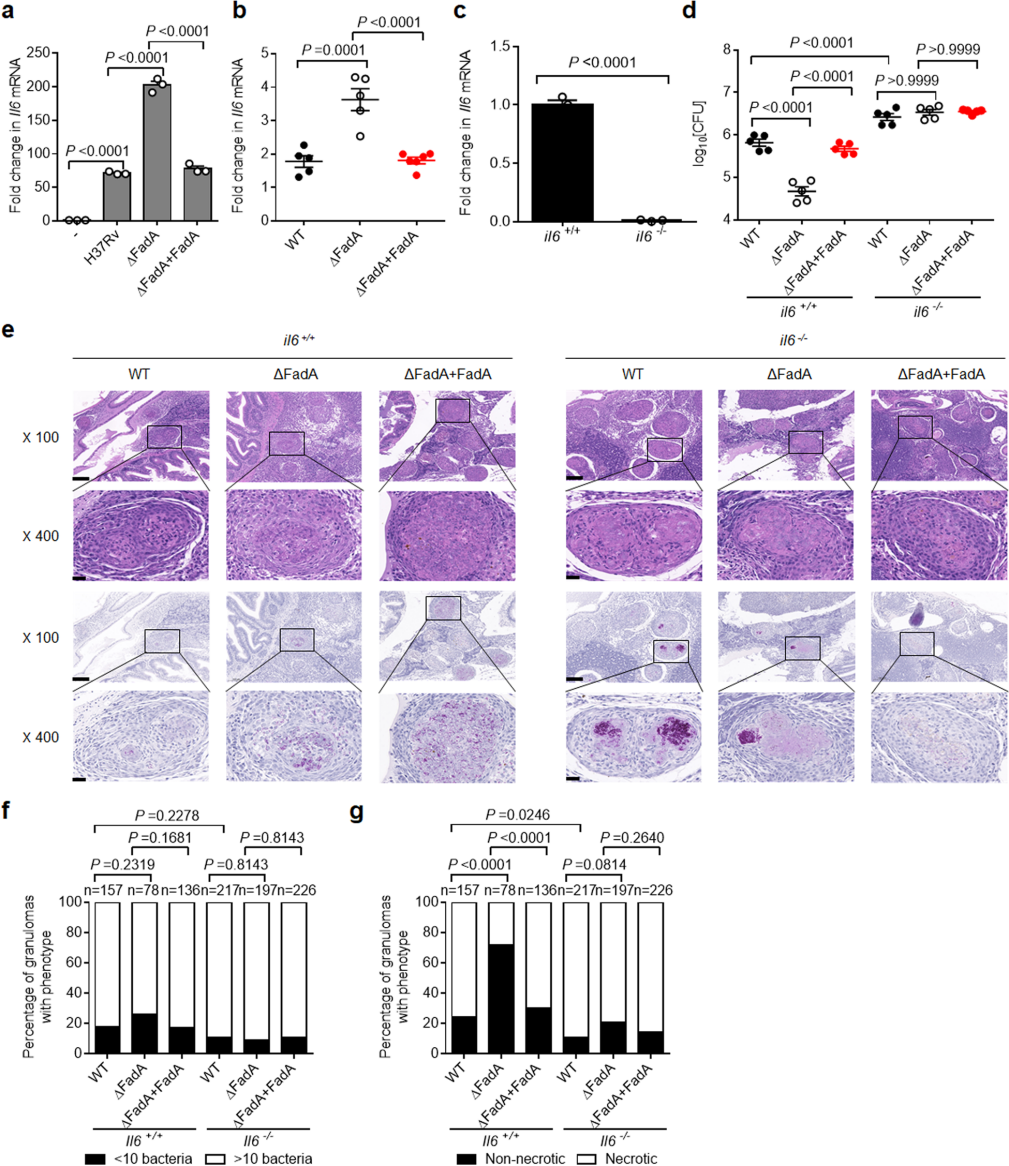
FadA inhibits host immune responses. **a** qPCR analysis of *Il6* mRNA from peritoneal macrophages infected with H37Rv, H37RvΔFadA, or H37Rv(ΔFadA + FadA) strains for 4 h (MOI = 1) (mean ± SEM). **b** qPCR analysis of *Il6* mRNA from adult zebrafish intraperitoneally infected with roughly 200 CFU per fish of WT, ΔFadA, or ΔFadA + FadA *M. marinum* strains for 14 days (mean ± SEM of *n* = 5). **c** qPCR analysis of *Il6* mRNA from *il6*^*+/+*^ and *il6*^*−/−*^ zebrafish (mean ± SEM). The Cas9/gRNA system was employed to generate IL-6 knockout zebrafish, which were constructed at the China Zebrafish Resource Center (CZRC) as described previously. **d**–**g** *il6*^*+/+*^ and *il6*^*−/−*^ mutant adult zebrafish were intraperitoneally infected with approximately 200 CFU of WT, ΔFadA, or ΔFadA + FadA *M. marinum* strains for 14 days. Bacterial titers (**d**; mean ± SEM of *n* = 5 fish infected for 14 days), histopathology (**e**; representative of one experiment with at least three independent replicates; scale bar, 100 μm (top) and 20 μm (bottom)), and comparison of granulomas between WT, ΔFadA, or ΔFadA + FadA *M. marinum*-infected *il6*^*+/+*^ and *il6*^*−/−*^ adult zebrafish scored for *M. marinum* burden as less than 10 or 10 or more bacteria (**f**) or percentage of necrotic granulomas in each fish (**g**) were assessed as described previously. “*n*” was the total number of granulomas for each strain infected fish. Total number of zebrafish analyzed: five (WT/*il6*^*+/+*^), five (ΔFadA/*il6*^*+/+*^), five (ΔFadA+FadA/*il6*^*+/+*^), five (WT/*il6*^*−/−*^), five (ΔFadA/*il6*^*−/−*^), five (ΔFadA + FadA/*il6*^*−/−*^). Data in **a**–**g** represent one experiment with at least three independent replicates. One-way ANOVA with Bonferroni’s multiple comparisons test (**a**, **b**, **d**), two-tailed unpaired Student’s *t-*test (**c**) and Fisher’s exact test (**f**, **g**) were used for statistical analysis.

To investigate the mechanism by which FadA enhances the survival of mycobacteria and granuloma progression, we examined whether FadA had any effect on the expression of proinflammatory cytokines. Peritoneal macrophages were infected with H37Rv, H37RvΔFadA, or H37Rv(ΔFadA + FadA) strain, and the expression of *Il1b*, *Il6*, *Il12b*, and *Tnf*α was analyzed by quantitative real-time polymerase chain reaction (qRT-PCR). Deletion of FadA in H37Rv markedly increased the expression of *Il6*, but not that of other cytokines in macrophages, which was also upregulated in the adult zebrafish infected with ΔFadA *M. marinum* compared with the levels in WT and FadA-complemented strains (Fig. 3a, b; Supplementary Fig. S4).

IL-6 was previously reported to be essential for anti-TB immunity^26^. Given that FadA enhances the survival of mycobacteria in tuberculous granulomas, and that FadA suppresses the expression of *Il6*, we next examined the functional relevance of IL-6 for the survival of mycobacteria in vivo. We generated an *il6* deletion mutant zebrafish allele using CRISPR/Cas9 genome editing mutagenesis^27^ (Supplementary Fig. S5; Fig. 3c). Deletion of *il6* markedly increased *M. marinum* burden compared with that in WT zebrafish (Fig. 3d), which is consistent with the protective immunity conferred by IL-6 against *M. tuberculosis* infection in mice^26^. Interestingly, deletion of the *il6* gene increased the proportion of necrotic granulomas compared with that in WT zebrafish (Fig. 3e–g), suggesting a previously unidentified role of IL-6 in the inhibition of tuberculous granuloma necrosis. Moreover, the reductions in the burden and rate of granuloma necrosis in WT fish infected with the *M. marinum* (ΔFadA) mutant compared with the levels in the WT *M. marinum* or *M. marinum* (ΔFadA + FadA) strain were not found in *il6*-deficient mutant zebrafish (Fig. 3d–g).

In parallel with this, we evaluated the effects of IL-6 on granuloma formation in C3HeB/FeJ mice infected with H37Rv. The C3HeB/FeJ mice were infected with H37Rv, H37RvΔFadA, or H37Rv(ΔFadA + FadA) strain, and then treated with a neutralizing anti-IL-6 or isotype-matched control monoclonal Ab (mAb) (Supplementary Fig. S6a). Neutralization of IL-6 markedly increased the bacterial burden and histological score of lesions in the lungs of C3HeB/FeJ mice infected with H37Rv (Supplementary Fig. S6b–d). Furthermore, treatment with anti-IL-6 mAb eliminated the reductions in bacterial burden and pathological score seen in H37RvΔFadA-infected mice compared with those in H37Rv or H37Rv(ΔFadA + FadA) control strain (Supplementary Fig. S6b–d). Together, these results demonstrate that increased host IL-6 mediates the enhanced killing of H37RvΔFadA.

### FadA suppresses IL-6 through H3K9Ac

**Fig. 4:**
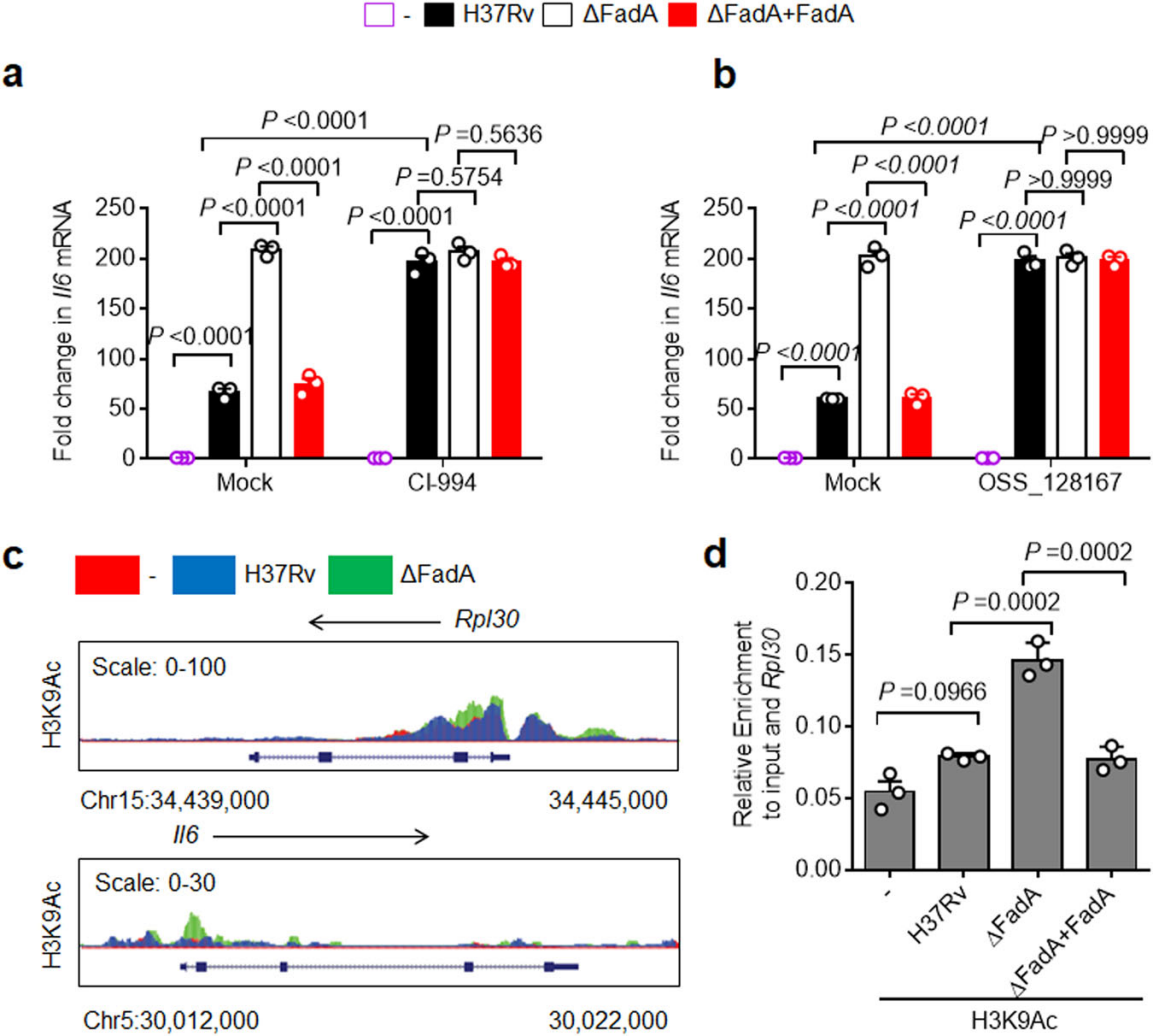
FadA suppresses IL-6 through H3K9Ac. **a** qPCR analysis of *Il6* mRNA from control or histone deacetylase (HDAC) 1–3 inhibitor CI-994-pretreated peritoneal macrophages infected with H37Rv, H37RvΔFadA, or H37Rv(ΔFadA + FadA) strains for 4 h (MOI = 1) (mean ± SEM). **b** qPCR analysis of *Il6* mRNA from control or OSS_128167, a selective inhibitor that increases the acetylation of H3K9-pretreated peritoneal macrophages infected with H37Rv, H37RvΔFadA, or H37Rv(ΔFadA + FadA) strains for 4 h (MOI = 1) (mean ± SEM). **c** ChIP-seq analysis of histone H3 acetylation at the lysine 9 residue (H3K9Ac) for the *Il6* promoter of peritoneal macrophage infected with H37Rv or H37RvΔFadA strains for 4 h (MOI = 1) with SimpleChIP Enzymatic Chromatin IP Kit. **d** ChIP-qPCR (mean ± SEM) analysis of H3K9Ac for the *Il6* promoter of peritoneal macrophage infected with negative control, H37Rv, H37RvΔFadA, or H37Rv(ΔFadA + FadA) strains for 4 h (MOI = 1). Data in **a**, **b**, **d** represent one experiment with at least three independent replicates. One-way (**d**) or two-way (**a**, **b**) ANOVA with Bonferroni’s multiple comparisons test were used for statistical analysis.

We next investigated the mechanism underlying the suppression of host IL-6 by mycobacterial FadA. There is accumulating evidence that the acetylation of histone proteins plays an important role in reversible gene expression changes^28–31^. From the BIOGPS database, we found that the expression of histone deacetylases (HDAC)1–3 was higher than that of other HDACs in macrophages (BioGPS). To investigate whether FadA regulates *Il6* expression through modulation of host histone acetylation, peritoneal macrophages were pretreated with CI-994, an inhibitor of HDAC1-3^32^, and infected with H37Rv, ΔFadA, or H37Rv(ΔFadA + FadA) strain. Treatment with CI-994 markedly induced the transcription of *Il6* in macrophages infected with H37Rv, but not the control genes (Fig. 4a; Supplementary Fig. S7a, b), suggesting a previously unidentified role of CI-994-sensitive histone acetylation in the regulation of *M. tuberculosis*-induced proinflammatory responses. The inhibitory effect of FadA on the expression of *Il6* in macrophages infected with H37Rv or H37Rv(ΔFadA + FadA) strain was abrogated by the treatment with CI-994 (Fig. 4a). Then, we pretreated macrophages with OSS_128167, a selective inhibitor that increases the acetylation of H3K9^33^, and infected them with the same strains. The data further confirmed that FadA suppresses *Il6* directly by modifying the acetylation of H3K9 (Fig. 4b**)**.

Lysine acetylation on the tails of histone H3 regulates cytokine-specific transcription during the innate immune response^34^. Our chromatin immunoprecipitation-sequencing (ChIP-seq) analysis of histone H3 acetylation at lysine 9 (H3K9Ac), a histone marker associated with active transcription, showed that the deletion of FadA led to increased enrichment of H3K9Ac surrounding host genome transcription start sites (TSSs) in macrophages infected with H37Rv (Supplementary Fig. S8). Compared with the case for the constitutively active *Rpl30* locus, deletion of FadA markedly increased the amount of H3K9Ac modification in the *Il6* promoter in H37Rv-infected macrophages (Fig. 4c, d), suggesting that FadA may inhibit *M. tuberculosis*-induced expression of *Il6* through reducing the level of H3K9Ac.

### FadA reduces acetyl-CoA

**Fig. 5:**
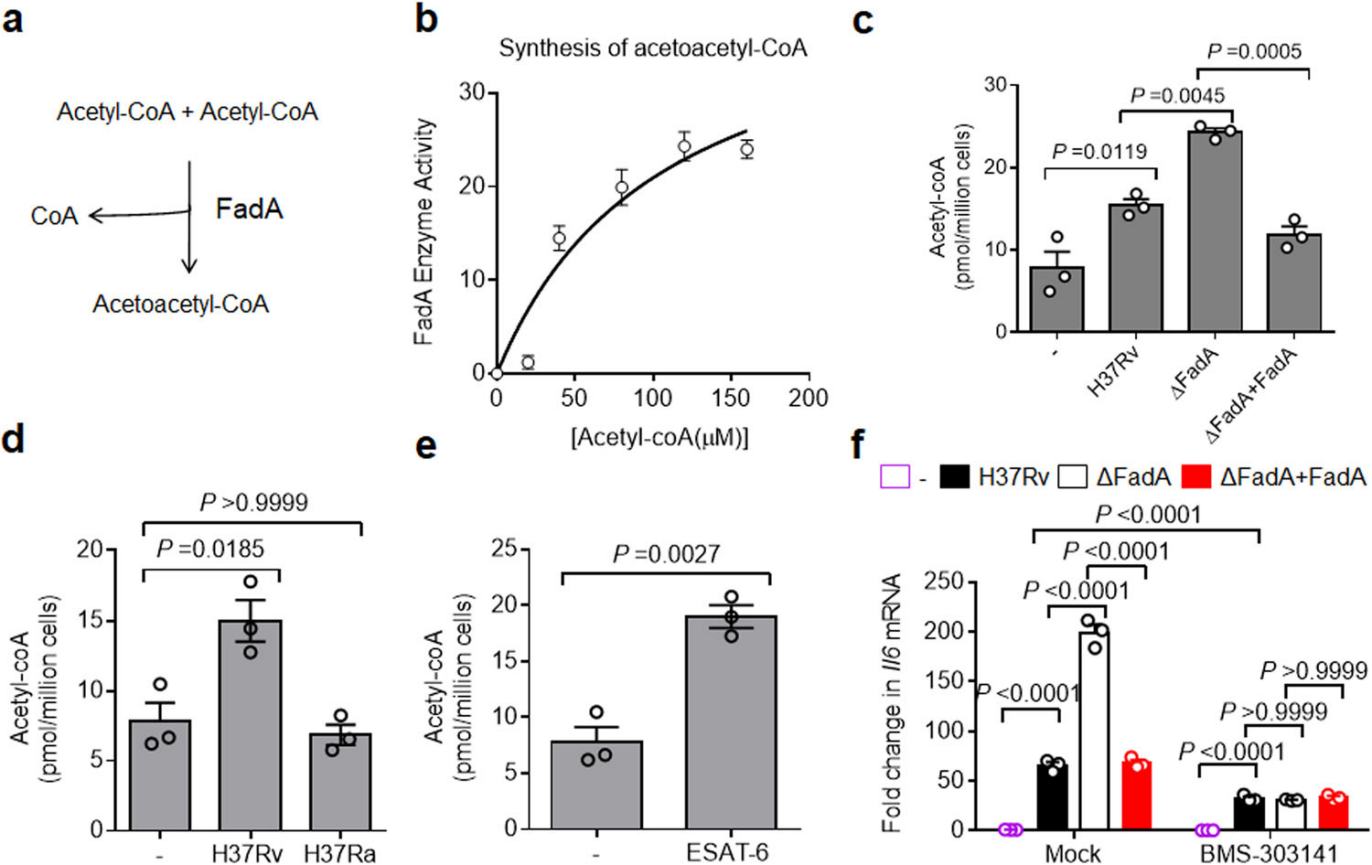
FadA reduces acetyl-CoA. **a** Diagram of the acetyl-CoA acetyltransferase activity of FadA. **b** In vitro two-substrate steady-state kinetic assay of acetyltransferase activity of recombinant FadA protein. **c** Acetyl-CoA level assay of peritoneal macrophages infected with H37Rv, H37RvΔFadA or H37Rv(ΔFadA + FadA) strains for 4 h (MOI = 1) (mean ± SEM). **d** Acetyl-CoA level assay of peritoneal macrophages infected with H37Rv or H37Ra for 4 h (MOI = 1) (mean ± SEM). **e** Acetyl-CoA level assay of peritoneal macrophages stimulated with recombinant ESAT-6 protein (5 μg/mL) for 4 h (mean ± SEM). **f** qPCR analysis of *Il6* mRNA from control or ATP-citrate lyase (ACL) inhibitor BMS-303141-pretreated peritoneal macrophages infected with H37Rv, H37RvΔFadA, or H37Rv(ΔFadA+FadA) strains for 4 h (MOI = 1) (mean ± SEM). Data in **b**–**f** are representative of one experiment with at least three independent biological replicates; each circle represents one technical repeat. One-way (**c**, **d**) or two-way (**f**) ANOVA with Bonferroni’s multiple comparisons test and two-tailed unpaired Student’s *t-*test (**e**) were used for statistical analysis.

Histone acetylation utilizes acetyl coenzyme A (acetyl-CoA) as a substrate^35,36^. According to the three-dimensional structure of FadA (PDB code: 4B3H)^37^, FadA appears to be an acyl-CoA thiolase, a key enzyme for the fourth step of reaction in the fatty acid β-oxidation pathway involving *M. tuberculosis*, which can catalyze the reversible thiolytic cleavage of 3-ketoacyl-CoA to acyl-CoA and acetyl-CoA^38^. However, the conserved domain alignment analysis revealed FadA to be an acetyl-CoA acetyltransferase that can catalyze the synthesis of acetoacetyl-CoA (AcAcCoA) from two molecules of acetyl-CoA^38^ (Fig. 5a). To examine the acetyl-CoA acetyltransferase activity of FadA, we expressed FadA as a C-terminally His6-tagged protein from *Escherichia coli* (*E. coli*) and purified using immobilized metal ion affinity chromatography (Supplementary Fig. S9a). The purified FadA was assayed for synthesis activity with acetyl-CoA as the substrate to yield AcAcCoA and coenzyme A at pH 8.1^39^. A two-substrate steady-state kinetic assay confirmed that the addition of FadA led to the consumption of acetyl-CoA in solution (Fig. 5b), suggesting that FadA may function as an acetyl-CoA acetyltransferase. Consistent with this finding, macrophages infected with H37RvΔFadA had a much higher level of acetyl-CoA in their cytoplasm than those infected with H37Rv or H37Rv(ΔFadA + FadA) strain (Fig. 5c). Intriguingly, peritoneal macrophages (Fig. 5d) or PMA-differentiated THP-1 cells^40–42^ responded to virulent *M. tuberculosis* strain H37Rv as well as its virulence factor ESAT-6, but not to its avirulent counterpart H37Ra (Fig. 5e), to increase the acetyl-CoA level, which drives the differentiation of foamy macrophages for the provision of nutrients^40–42^. Together, our results suggest that FadA may act as an acetyl-CoA acetyltransferase that restrains host acetyl-CoA levels in *M. tuberculosis-*infected macrophages.

To determine whether FadA inhibits the expression of *Il6* by reducing cytosolic acetyl-CoA, we treated macrophages with BMS-303141, an inhibitor of ATP-citrate lyase (ACL), which is the enzyme that catalyzes the conversion of citrate to acetyl-CoA^43^. Treatment with BMS-303141 markedly reduced the H37Rv-induced expression of *Il6*, suggesting that acetyl-CoA may enhance the proinflammatory responses to *M. tuberculosis* infection (Fig. 5f). Furthermore, the inhibition of ACL by BMS-303141 ablated the inhibitory effect of FadA on the expression of *Il6* (Fig. 5f). Taken together, these results suggest that FadA may suppress the anti-TB immunity by reducing cytoplasmic acetyl-CoA.

### FadA functions through its acetyltransferase activity

**Fig. 6:**
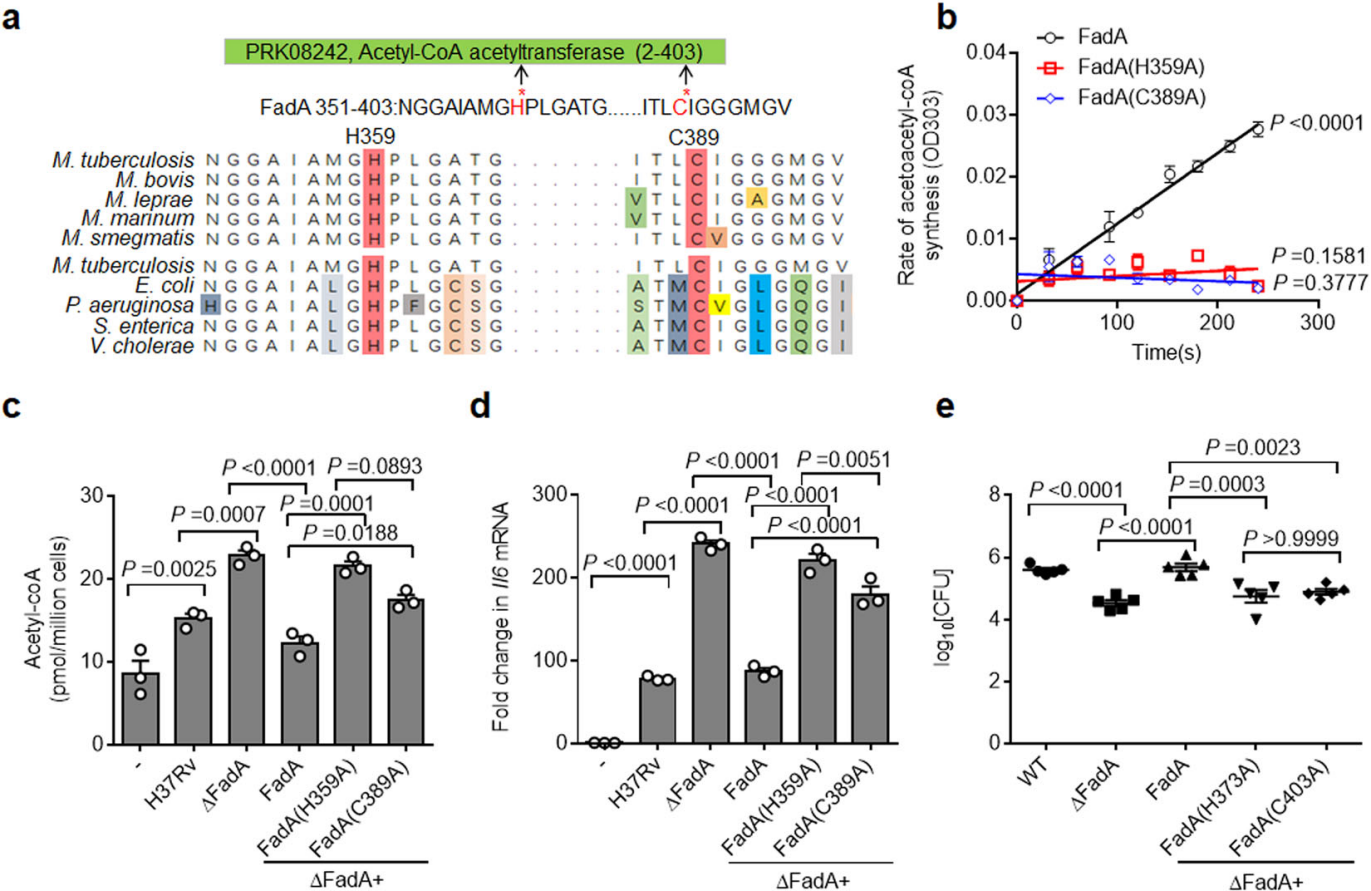
FadA functions through its acetyltransferase activity. **a** Alignment of H359 and C389 sites of FadA in mycobacteria and bacteria. **b** In vitro two-substrate steady-state kinetic assay of acetyltransferase activity of recombinant FadA and FadA-Mut (FadA(H359A), FadA(C389A)) protein. **c** Assay of the acetyl-CoA level of peritoneal macrophages infected with H37Rv, H37RvΔFadA, H37Rv(ΔFadA + FadA), H37Rv(ΔFadA + FadA(H359A)), and H37Rv(ΔFadA + FadA(C389A)) strains for 4 h (MOI = 1) (mean ± SEM). **d** qPCR analysis of Il6 mRNA of peritoneal macrophages infected with H37Rv, H37RvΔFadA, H37Rv(ΔFadA + FadA), H37Rv(ΔFadA + FadA(H359A)), and H37Rv(ΔFadA + FadA(C389A)) strains for 4 h (MOI = 1) (mean ± SEM). **e** Adult zebrafish were intraperitoneally infected with roughly 200 CFU of WT, ΔFadA, ΔFadA + FadA, ΔFadA + FadA(H373A), and ΔFadA + FadA(C403A) *M. marinum* strains for 14 days. Histopathology was assessed in the whole fish by bacterial titers (mean ± SEM of *n* = 5 fish infected for 14 days). Total number of zebrafish analyzed: five (WT), five (ΔFadA), five (ΔFadA + FadA), five (ΔFadA + FadA(H373A)), five (ΔFadA + FadA(C403A)). Data in **b**–**e** represent one experiment with at least three independent replicates. One-way ANOVA with Bonferroni’s multiple comparisons test (**c**–**e**) was used for statistical analysis.

Analysis of the three-dimensional structure of FadA in *M. tuberculosis*^37^ identified two key enzymatic sites, H359 and C389, which are evolutionarily conserved in FadA isozymes from mycobacteria and common pathogenic bacteria (Fig. 6a). The H359 and C389 residues of FadA were mutated individually to alanine, and FadA(H359A) and FadA(C389A) were expressed as recombinant proteins from *E. coli* (Supplementary Fig. S9a). Mutation of either site abrogated the acetyl transfer activity of FadA (Fig. 6b), suggesting that H359 and C389 are essential for the acetyltransferase activity of FadA. To further investigate whether FadA reduces cytoplasmic acetyl-CoA via H359 or C389, the H37RvΔFadA strain was complemented with FadA(H359A) or FadA(C389A) to generate the H37Rv(ΔFadA + FadA(H359A)) and H37Rv(ΔFadA + FadA(C389A)) strains (Supplementary Fig. S9b), and the conserved H373 and C403 residues of FadA in *M. marinum* were mutated individually to alanine, and the ΔFadA + FadA(H373A) and ΔFadA + FadA(C403A) *M. marinum* strains were constructed based on *M. marinum* (ΔFadA) (Supplementary Fig. S9c). The secretion and total synthesis of FadA or various FadA mutants in *M. tuberculosis* and *M. marinum* under aeration and hypoxia were examined by western blotting analysis of cell lysates and culture supernatants. The data showed that the secretion and synthesis of FadA mutants were induced by hypoxia at similar levels to WT FadA (Supplementary Fig. S9d, e). Reconstitution of H37RvΔFadA with FadA, but not the FadA(H359A) or FadA(C389A) mutants, markedly decreased the level of acetyl-CoA in the *M. tuberculosis*-infected macrophages (Fig. 6c). These results demonstrate that FadA enzymatically reduces host acetyl-CoA level through H373 and C403 residue-dependent acetyltransferase activity.

To examine whether the enzymatic activity of FadA is also required for its inhibitory effect on the expression of *Il6*, primary peritoneal macrophages were infected with H37Rv(ΔFadA + FadA), H37Rv(ΔFadA + FadA(H359A)), or H37Rv(ΔFadA + FadA(C389A)) strain individually, and the mRNA level of *Il6* was examined. The results demonstrated that neither FadA(H359A) nor FadA(C389A) inhibited the expression of *Il6* compared with WT FadA (Fig. 6d), indicating that the acetyltransferase activity is essential for FadA to inhibit the host proinflammatory response. Moreover, the mRNA level of *Il6* in all macrophages infected with different H37Rv strains was increased by the treatment of CI-994 (Supplementary Fig. S10a), but decreased by BMS-303141 (Supplementary Fig. S10b). Together, these results suggest that the inhibitory effect of FadA on the expression of *Il6* may require its acetyltransferase activity.

To determine whether FadA promotes the progression of tuberculous granulomas via the two key enzymatic sites, the ΔFadA + FadA, ΔFadA + FadA(H373A), or ΔFadA + FadA(C403A) *M. marinum* strains were used to infect adult zebrafish. First, the mRNA level of *Il6* in the adult zebrafish infected with each *M. marinum* strain for 14 days was examined, and we found that neither FadA(H373A) nor FadA(C403A) inhibited the expression of *Il6* compared with WT FadA (Supplementary Fig. S10c). Second, we infected adult zebrafish with the ΔFadA + FadA, ΔFadA + FadA(H373A), or ΔFadA + FadA(C403A) *M. marinum* strains and demonstrated that reconstitution of *M. marinum* (ΔFadA) with FadA but not the FadA(H373A) or FadA(C403A) mutant led to a much higher bacterial burden and markedly increased the fraction of necrotic granulomas in adult zebrafish (Fig. 6e; Supplementary Fig. S10d–f). This indicates that FadA promotes the in vivo survival of mycobacteria and necrosis of tuberculous granulomas through its acetyltransferase activity.

### Targeting acetyl-CoA for TB therapy

**Fig. 7:**
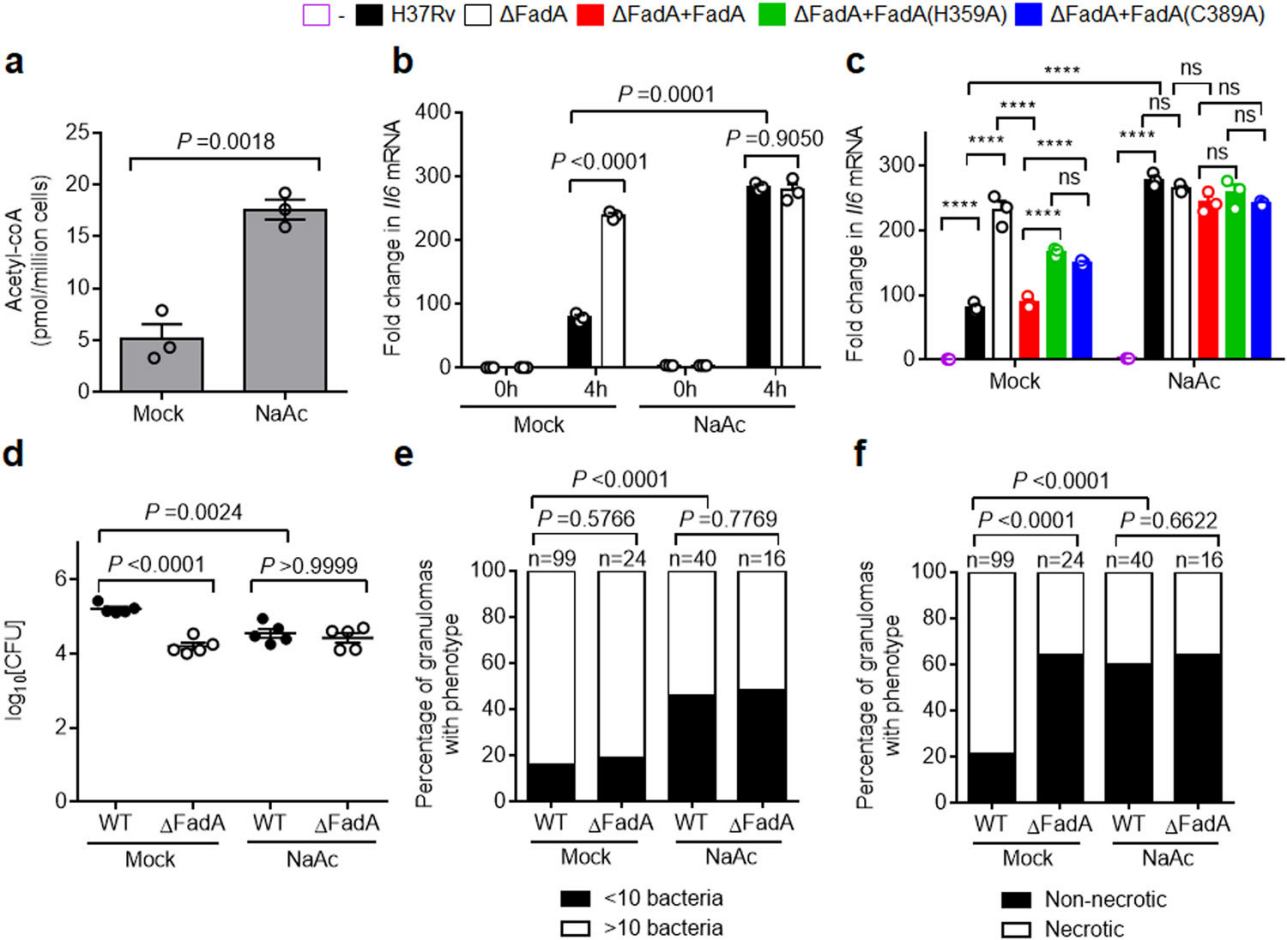
Targeting acetyl-CoA alleviates pathogenesis of mycobacteria. **a** Acetyl-CoA level assay of peritoneal macrophages supplied with 2 mM NaAc for 24 h. **b** qPCR analysis of *Il6* mRNA from control or 2 mM NaAc-supplemented peritoneal macrophages infected with H37Rv or H37RvΔFadA strains for 0 or 4 h (MOI = 1) (mean ± SEM). **c** qPCR analysis of *Il6* mRNA from control or 2 mM NaAc-supplemented peritoneal macrophages infected with H37Rv, H37RvΔFadA, H37Rv(ΔFadA + FadA), H37Rv(ΔFadA + FadA(H359A)), and H37Rv(ΔFadA + FadA(C389A)) strains for 4 h (MOI = 1) (mean ± SEM). Data in **a**–**c** are representative of one experiment with at least three independent biological replicates; each circle represents one technical repeat. Bar charts show means. **d**–**f** Adult zebrafish were intraperitoneally infected with roughly 200 CFU of WT or ΔFadA *M. marinum* strains for 14 days. NaAc was injected intraperitoneally to a final concentration of 2 mM immediately after infection and reinjected every 3 days. Histopathology was assessed in the whole fish by bacterial titers (**d**; mean ± SEM of *n* = 5 fish infected for 14 days), comparison of granulomas between different *M. marinum*-infected adult zebrafish scored for *M. marinum* burden as less than 10 or 10 or more bacteria (**e**), or percentage of necrotic granulomas in each fish (**f**). A total number of zebrafish analyzed: five (WT/Mock), five (ΔFadA/Mock), five (WT/NaAc), and five (ΔFadA/NaAc). Data in **d**–**f** represent one experiment with at least three independent replicates. Two-tailed unpaired Student’s *t-*test (**a**, **b**), One-way (**d**) or two-way (**c**) ANOVA with Bonferroni’s multiple comparisons test and Fisher’s exact test (**e**, **f**) were used for statistical analysis (ns, not significant; ****P < 0.0001).

Host-directed therapy (HDT) has emerged as an attractive strategy for treating TB, especially drug-resistant TB, by targeting host pathways to increase anti-TB immunity^44^. Since mycobacterial FadA protein promotes the survival of pathogenic mycobacteria and necrosis of tuberculous granulomas through manipulating host fatty acid metabolism, we next addressed the therapeutic effect of targeting host fatty acid metabolism in adult zebrafish. Acetyl-CoA is known to be produced from acetate by acetyl-CoA synthetase^45^. Acetate supplementation significantly increased the level of acetyl-CoA in peritoneal macrophages (Fig. 7a), indicating that acetate is an inducer of acetyl-CoA. Consistent with this, treatment with acetate also markedly enhanced the expression of *Il6* in H37Rv-infected macrophages (Fig. 7b). Moreover, the addition of acetate abrogated the inhibitory effect of FadA on the expression of *Il6* in macrophages infected with H37Rv (Fig. 7b). Similarly, the differential inhibition of WT FadA and its inactive mutants FadA(H359A) or FadA(C389A) on the expression of *Il6* in H37Rv-infected macrophages was not observed following the addition of acetate (Fig. 7c). Finally, treatment of acetate markedly reduced the bacterial burden and pathological lesions in the zebrafish infected with *M. marinum* (Fig. 7d–f; Supplementary Fig. S11). These results suggest that targeting host fatty acid metabolism may provide an adjunctive therapy for mycobacterial infections.

## Discussion

Adaptation to hypoxia, evasion of anti-TB immunity, and utilization of limited host-derived nutrients are major challenges for mycobacteria to establish granulomas and maintain successful persistent infection in the host. In this study, we demonstrate that hypoxia specifically induces FadA, which functions as an immunomodulatory factor to suppress host pro-inflammatory responses. Deletion of FadA attenuates the survival of mycobacteria in vivo through enhancing the host pro-inflammatory responses and preventing granuloma necrosis. FadA-mediated conversion of acetyl-CoA to AcAcCoA may simultaneously provide the substrate for the synthesis of ketone bodies and accumulation of lipid bodies, driving acquisition of the foamy phenotype by host macrophages^40–42^. Thus, FadA appears to be induced by hypoxia to suppress host anti-TB immunity and possibly enhance the accumulation of host-derived nutrients, thus economically facilitating the adaptation of mycobacteria to hypoxic granuloma for persistent infection.

One unusual feature of pathogenic mycobacteria is their five paralogous ESX-1 type VII secretion systems, which transport their effector proteins across the hydrophobic and highly impermeable cell walls into the cytoplasm of host cells^10^. We have profiled the secretion of H37Rv under hypoxia and found that hypoxia either increased or decreased the secretion of different H37Rv proteins, indicating the adaptation of this species to hypoxia through trimming transportation via secretion systems. Interestingly, most proteins that are secreted more by hypoxia are related to fatty acid metabolism, encoded by genes that are already relatively “overrepresented” in the *M. tuberculosis* genome^20^. The H37Rv protein with the most significant increase in the secretion, FadA, acts as an acetyltransferase that interferes with the host fatty acid metabolism. The function of FadA to convert host acetyl-CoA to AcAcCoA represents an unusual mechanism in host–microbe interactions, and research may expand to other fatty acid metabolism-related H37Rv secreted proteins including Rv0860 and Rv3774, which are highly expressed under hypoxia.

Pathogenic mycobacteria have evolved to interfere with host innate immune responses to establish successful infection^46–48^. From qRT-PCR validation, we confirmed that FadA could inhibit the expression of the proinflammatory cytokine *Il6*. It has been shown that IL-6 has a crucial role in protecting against infection by high doses of intravenously delivered *M. tuberculosis*^26^, but is not required for the generation of specific immunity to infection by low-dose aerosol-delivered *M. tuberculosis* in C57BL/6J mice^49^. Here, our study indicates that the inhibition of IL-6 by genetic deletion or antibody neutralization promotes the progression of granuloma as well as the survival of mycobacteria in adult zebrafish infected with *M. marinum* or C3HeB/FeJ mice infected with H37Rv. To our knowledge, this is the first study to demonstrate an inhibitory role of IL-6 in the progression of mycobacterial granuloma. Furthermore, our data show that FadA promotes the survival of mycobacteria and the progression of tuberculous granuloma necrosis through inhibiting IL-6. *M. tuberculosis* infection usually activates the MAP kinase or NF-κB pathway to induce the expression of proinflammatory cytokines such as *Il6*^46–48^. However, activation of the MAP kinase or NF-κB pathway is not inhibited by FadA (data not shown). Histone modifications such as hyperacetylation of histone H3 are known to be active markers of gene expression and are often associated with the ongoing transcription of cytokines^29,50^. Our data demonstrate that FadA inhibits the acetylation of H3K9 at the *Il6* promoter of H37Rv-infected macrophages, and suppresses the expression of the *Il6* gene through modulating histone acetylation, suggesting a previously unidentified but important role of histone acetylation in the regulation of *M. tuberculosis*-induced proinflammatory responses. The histone code hypothesis suggests that multiple histone modifications act in a combinatorial fashion to specify distinct chromatin states^51^. Therefore, more acetylation positions of histones in macrophages infected with the H37Rv or H37RvΔFadA strains need to be detected and analyzed together.

Most mycobacterial secreted proteins are believed to inhibit host innate immune responses through interacting with host immune signaling proteins^46–48,52^. However, recent studies have indicated that the metabolic regulation of host macrophages determines pathogen growth or containment^53–55^. Successful import and utilization of host-derived fatty acids are required for the physiology and pathogenesis of *M. tuberculosis*^21,56,57^. Here, we found that FadA acted as an acetyl-CoA acetyltransferase to reduce the cytoplasmic level of acetyl-CoA. Acetyl-CoA, as a substrate for histone acetylation^35,36^, has been found to increase histone H3K9 acetylation to trigger the expression of proinflammatory cytokines in H37Rv*-*infected macrophages. It has been reported that the virulent H37Rv protein ESAT-6 modulates mitochondrial pyruvate transporter activity to markedly increase the total cellular level of acetyl-CoA^40–42^. However, the excessive accumulation of acetyl-CoA may facilitate histone acetylation to increase the expression of proinflammatory cytokines and promote an effective immune response against mycobacteria. Simultaneously, FadA appears to counteract the ESAT-6-mediated increase in cytoplasmic acetyl-CoA to suppress histone acetylation-mediated anti-TB immunity. We further speculate that the production of AcAcCoA by FadA may simultaneously promote the production of AcAcCoA as the substrate for the synthesis of ketone bodies and accumulation of lipid bodies, driving acquisition of the foamy phenotype by the host macrophages^40,42^. However, the exact mechanism underlying the regulation of FadA in lipid synthesis requires further study.

There is an urgent need to develop novel therapeutic approaches for the clinical treatment of TB, especially drug-resistant TB. HDT is emerging as an attractive strategy harnessing the host–microbe interaction to generate immune responses against *M. tuberculosis* instead of direct bactericidal effects^44^. Here, we demonstrate that acetate supplementation can increase the level of acetyl-CoA in peritoneal macrophages, and markedly reduce granuloma necrosis and mycobacterial burden in the adult zebrafish-*M. marinum* infection model. In addition, the three-dimensional structure of FadA has already been solved^37^, and two evolutionarily conserved enzymatic sites, H359 and C389, have been found to be essential for virulence. Using computer-assisted simulation, we have identified 80 compounds acting against the activity of FadA, and study of the effects of these compounds on the survival of mycobacteria and granuloma formation is currently ongoing. Together, our findings provide a novel HDT strategy for TB infection by targeting the interaction of FadA with the host fatty acid metabolic pathway.

In summary, our findings identify FadA as a previously unrecognized mycobacterial virulence factor that harnesses an unusual mechanism of integrating the adaptation to hypoxia with the inhibition of host anti-TB immunity and probably utilizes host-derived nutrients to facilitate the formation of the granuloma for persistent infection (Supplementary Fig. S12). The reduction in the host acetyl-CoA level by FadA to suppress anti-TB immunity is distinguished from the utilization of host-derived fatty acids as favored nutrient resources for *M. tuberculosis*^54,55,57^. Accordingly, the conversion of acetyl-CoA to AcAcCoA by FadA reduces the cytoplasmic level of acetyl-CoA in host cells to suppress the histone H3K9 acetylation-mediated expression of *Il6*, a factor not previously known to inhibit granuloma pathology. Therefore, the depletion of host acetyl-CoA by hypoxia-induced FadA represents an unusual strategy for *M. tuberculosis* under hypoxic conditions to intercept the host fatty acid metabolism to suppress anti-TB immunity and adapt to hypoxic granuloma for persistent infection. This provides insights for the design of effective approaches to eliminate TB or even drug-resistant TB infection through targeting the interface between mycobacteria and host fatty acid metabolism.

## Materials and methods

### Bacterial strains and cells

All strains used in this study are described in Supplementary Table S3. The *Mycobacterium tuberculosis* H37Rv, *Mycobacterium marinum* Aronson (BAA-535) strains were grown in Middlebrook 7H9 broth (Difco/Becton Dickson, Franklin Lakes, NJ) supplemented with 10% ADC (5% bovine serum albumin (BSA), 2% dextrose, 5% catalase) and 0.05% Tween-80 (Sigma) or Middlebrook 7H10 agar (Difco) supplemented with 10% ADC and antibiotic supplements as required. The antibiotics used were 50 μg/mL or 75 μg/mL hygromycin B (Thermo) and 50 μg/mL kanamycin (Sigma).

Primary peritoneal macrophages were harvested as described previously^58^. Briefly, C57Bl/6 mice were intraperitoneally injected with 2 mL of 4% Brewer thioglycollate medium (Sigma). After 3 days, the mice were sacrificed by cervical dislocation, and cells were isolated by flushing the peritoneal cavity with 10 mL of RPMI-1640 (Thermo) medium per mouse. Cells were seeded in a 24-well dish, and non-adherent cells were removed by extensive washing with RPMI-1640. The adherent peritoneal macrophages were cultured in RPMI-1640 medium supplemented with 10% (v/v) heat-inactivated fetal bovine serum (FBS, Thermo) and 100 U/mL penicillin and streptomycin (Thermo) and used for subsequent experiments. All the cells were routinely tested for contamination by mycoplasma.

### Bacterial cultures

Wayne experiments in H37Rv were performed as described previously^18^. In brief, conical screw-capped Nephelo flasks with 20 mm side arms and flat bases (Wheaton Scientific Products, Millville, NJ) were used to culture bacteria. H37Rv was grown to mid-log phase (OD590 ≈ 0.4, probably 2.5 × 10^8^ colony-forming unit (CFU)/mL). For aerobic conditions, 200 mL of medium was inoculated with 2 mL of the culture and incubated at 37 °C on a magnetic stirrer set rotating at 180 rpm. Simultaneously, 400 mL of medium was inoculated with 4 mL of identical culture in a tightly capped flask, placed on a tissue culture magnetic stirrer set at 70 rpm, and incubated at 37 °C to avoid perturbation of the surface. Samples for the quantitative proteomic assay were taken after 14 days of vigorous aeration of cultures (aeration) and hypoxic cultures (hypoxia).

For western blotting or qPCR detection of bacterial cultures, mycobacteria were grown to the mid-log phase as before. For aerobic conditions, 4 mL of the protein-free liquid medium in the 20 mm screw-capped culture tubes were inoculated with 0.4 mL of the culture and incubated at 37 °C in a shaker-incubator set rotating at 180 rpm. Simultaneously, 8 mL of medium was inoculated with 0.8 mL of identical culture in the tightly capped tubes, placed on a shaker-incubator set rotating at 70 rpm, and incubated at 37 °C to avoid perturbation of the surface. Then 4 mL cultures under aeration or hypoxia after indicated days were individually collected and centrifuged to separate the culture supernatant and precipitated bacteria cell. For samples at day 0, identical mycobacteria cultures were added in the aeration or hypoxia culture tubes similarly, and the culture supernatant and precipitated bacteria cell were collected immediately.

H37Rv and *M. marinum* cultures were grown up to the mid-log phase. Aliquots of bacterial cultures were prepared in 20% glycerol and Middlebrook 7H9 medium and preserved at −80 °C. The CFU per milliliter of the stocks was titered by plating serial dilutions on Middlebrook 7H10 agar plates plus 10% ADC. These stocks were used for all subsequent infections of macrophages, zebrafish, and mice.

### Plasmids, reagents, and antibodies

Plasmids used in this study are described in Supplementary Table S3. Direct-Blot HRP anti-*E. coli* RNA Sigma 70 (SigA) antibody (663205) was purchased from BioLegend. Rabbit polyclonal to ESAT6 (ab45073) and polyclonal rabbit anti-Histone H3 (acetyl K9) antibody (ab4441, ChIP grade) were purchased from Abcam. The rabbit polyclonal antibody to FadA was generated by immunization of rabbits with the purified SUMO-FadA fusion protein, in collaboration with ABclonal Biotech, so as the isotype control antibody. InVivo Mab anti-mouse IL-6 (BE0046) and InVivo Mab rat IgG1 isotype control (BE0088) were purchased from BioXcell. Recombinant ESAT6 protein was from the laboratory stock. Enzymatic chromatin IP kit (magnetic beads) (9003) was from Cell Signaling Technology. Acetyl-Coenzyme A Assay Kit (MAK039) was from Sigma. ECL reagent (34075) was from Thermo.

### Quantitative comparative proteomic profiling of culture filtrate supernatant from H37Rv under aeration or hypoxia

The culture supernatant and collected cells were separated via filtration through a membrane with 0.22 μm pore (Millipore). The culture filtrate protein samples were prepared. Briefly, ProteoMiner™ Protein Enrichment Small-Capacity Kit was used following the manufacturer’s instructions. Then, the protein concentration was determined with a 2-D Quant kit, in accordance with the manufacturer’s instructions. For trypsin digestion, the supernatant was transferred to a new tube, reduced with DTT for 1 h at 37 °C, and alkylated with IAA for 45 min at room temperature in the dark. The protein was precipitated with prechilled acetone overnight at −20 °C, washed with acetone three times, and then redissolved in TEAB. Approximately, 200 μg of protein for each sample was digested with trypsin overnight at 37 °C. After trypsin digestion, the peptide was desalted using a Strata X C18 SPE column (Phenomenex) and vacuum-dried. The peptide was reconstituted in TEAB and processed in accordance with the manufacturer’s protocol for the 6-plex TMT kit. Peptides were dissolved, directly loaded onto a reversed-phase column packed in-house with 3-μm C18 beads (Reprosil-Pur C18-AQ, Dr. Maisch), and eluted with a linear gradient of solvent B on an EASY-nLC 1000 UPLC system. The resulting peptides were analyzed by Q Exactive (ThermoFisher) and subjected to NSI source, followed by tandem mass spectrometry (MS/MS) coupled online with UPLC. For MS scans, the m/z scan range was 350–1600 Da. The MS/MS data files were merged and transformed to an MGF file by using Proteome Discoverer (Version 1.3.0.339, Thermo). Peptide and protein identifications were performed using the Mascot search engine (Version 3.2). We collected the protein sequence of H37Rv from Uniprot. The complete list of identified peptides was then housed in an Excel file for the grouping of results into proteins and the calculation of ratios and coefficients of variation.

### Immunolocalization analysis

For immunohistochemistry (IHC), lung tissue from a TB patient or a lung cancer patient with pulmonary lobectomy and whole fish sections of *M. marinum* infected or uninfected adult zebrafish were investigated. Segments of lung tissues and whole fish were fixed in 10% buffered formalin and embedded in paraffin. The blocks were cut into 5 μm sections, and five noncontiguous sections were processed for IHC staining with anti-FadA polyclonal antibody at a 1:100 dilution and anti-rabbit secondary antibody labeled with HRP (Servicebio) at a 1:200 dilution, compared with anti-ESAT-6 polyclonal antibody at a 1:200 dilution, isotype polyclonal control antibody at a 1:100 dilution, anti-SigA antibody labeled with HRP at 1:100 dilution and acid-fast staining. Immunostaining was visualized with 3,3-diaminobenzidine (DAB, Servicebio) substrate. After counterstaining in hematoxylin, the sections were mounted and examined. The studies on clinical samples were conducted in accordance with ethical guidelines of the Institutional Review Board of Shanghai Pulmonary Hospital (SYXK2018-0033).

### Construction of *fadA*-modified mycobacterial strains

Gene knockouts of FadA in H37Rv and *M. marinum* were generated using allelic exchange and a specialized transducing phage, phAE87, as described previously^59^. In brief, upstream and downstream flanking regions were amplified using the primer pairs and cloned into the delivery vector pYUB854. Allelic exchange constructs were incorporated into the phAE87 phage and phagemid DNA was electroporated into electrocompetent *M. smegmatis* cells to obtain plaques from the transduced 7H10 agar plates. Specialized transducing phages were picked and amplified at 30 °C to generate high-titer mycobacteriophages. The desired phage was transduced into H37Rv or *M. marinum* to delete the FadA gene by specialized transduction. The transductants were plated on selective medium, 7H10 agar containing 10% ADC enrichment and 75 μg/mL hygromycin, and cultured at 37 °C for H37Rv, 30 °C for *M. marinum*. Hygromycin-resistant clones were isolated and analyzed by PCR and qPCR to confirm the deletion of target genes. Western blotting was used to confirm the deletion of FadA with anti-FadA polyclonal antibody at a 1:1000 dilution; anti-SigA monoclonal antibody at a 1:1000 dilution was used as the reference antibody. The complementation plasmid for the FadA mutant was generated by ligating the PCR products using FadA-specific primers (Supplementary Table S4) into the episomal vector, pVV16 with hsp60 promoter. The resulting plasmid was transformed into the ΔFadA strain and plated on a 7H10 plate containing kanamycin at 50 μg/mL. Positive integrants carrying the required insert were screened by colony PCR and validated by qPCR and immunoblot analyses.

### Growth curve determination

Mycobacteria were grown to mid-log phase in 7H9 broth with 10% ADC, 0.05% Tween 80, and antibiotics, as required. Growth curves for each strain were determined using a Bioscreen C Growth Curve Instrument (Labsystems Oy, Helsinki, Finland) and a honeycomb plate with 100 wells (Labsystems Oy). Briefly, 200 μL of each bacterial suspension, adjusted to a similar density, was added to each well and cultured with shaking at 37 °C for H37Rv and 30 °C for *M. marinum*. The optical density was measured at an absorbance of 590 nm every day. Hypoxic conditions were established by covering each culture with 50 μL of paraffin oil. Cultures were incubated at 37 °C for 14 days for H37Rv and 10 days for *M. marinum*. Three independent experiments were performed, each in triplicate.

### ChIP-seq and ChIP-qPCR analysis

ChIP was were performed with SimpleChIP Enzymatic Chromatin IP Kit, in accordance with the manufacturer’s instructions. In brief, primary peritoneal macrophages were stimulated with H37Rv or H37RvΔFadA strains for 4 h and were fixed for 10 min at 25 °C with 1% formaldehyde. After incubation, glycine was added to a final concentration of 0.125 M to quench formaldehyde. Subsequently, cells were lysed and chromatin was harvested and fragmented using enzymatic digestion followed by sonication. The chromatin was then subjected to immunoprecipitation with anti-H3K9Ac at 4 °C overnight and was incubated with protein G magnetic beads at 4 °C for 2 h. The immune complexes were washed and eluted in 150 μL of elution buffer. Elute DNA and input DNA were incubated at 65 °C to reverse the cross-linking. After digestion with proteinase K, DNA was purified with spin columns. The relative abundance of precipitated DNA fragments was analyzed by qPCR using SYBR Green PCR Master Mix and the enrichments were normalized to *Rpl30* promoter (Cell Signaling Technology). Primer pairs used for *Il6* ChIP-qPCR are listed in Supplementary Table S4.

### ChIP-seq data analysis

ChIP sample quality was assessed using a bioanalyzer (Agilent Technologies). ChIP-seq libraries were prepared in accordance with the Illumina TruSeq ChIP sample protocol. They were then pooled for deep sequencing using Illumina Hiseq Xten (2 × 150) platforms at the CAS-MPG Partner Institute for Computational Biology Omics Core (Shanghai, China). Raw read quality was evaluated with FastQC (http://www.bioinformatics.babraham.ac.uk/projects/fastqc/v0.11.5). Adapter sequences and read sequences on both ends with Phred quality scores below 30 were trimmed. Trimmed reads were then mapped with the Bowtie2 algorithm (version 2.3.0) to the mouse genome (version mm10). Reads that failed to be mapped or that mapped at several locations (as identified by the XS tag set by Bowtie2) were removed. Read duplicates were identified and removed using Picard’s Mark Duplicates (http://broadinstitute.github.io/picard). Peak calling was performed using MACS2 (version 2.1.1). For all experiments, MACS2-defined peaks from all conditions (–, H37Rv and ΔFadA) were merged into a unique non-overlapping set of 21,708 peaks for H3K9AcIP (filtered for *P* value < 0.00001). Genome-wide signal coverage tracks at every bp were also computed by MACS2 and visualized in the UCSC Genome Browser. Peaks were annotated to the nearest genes within 2 kb of the TSS using ngsplot (version 2.61). Peaks overlapping by at least 1nt with unique gene model promoters (±2 kb of each unique gene model transcription starting site) were considered as being located at promoters. De novo motif searches of ChIP-seq peaks were performed using MEME.

### Purification of FadA and its derivative mutants

The construction and purification of H37Rv FadA recombinant protein and its derivative mutants were performed as described previously. pET28a-FadA, pET28a-FadA(H359A), and pET28a-FadA(C389A) plasmids are described in Supplementary Table S3.

### In vitro two-substrate steady-state kinetics assay of acetyltransferase activity

Acetyltransferase activity was measured following the methods of Thompson et al.^60^, and Middleton^61^. In brief, assay mixtures containing 100 mM Tris-HCl, pH 8.1, 25 mM MgCl_2_, 50 mM-KCI, and 0–200 M acetyl-CoA (Sigma) were preincubated at 30 °C for 5 min. The background rate of acetyl transfer was measured. Reactions were initiated by the addition of FadA or its derivative mutants (250 nM) to the assay mixture. The formation of acetoacetyl-CoA-Mg^2^-enolate was followed at 303 nm for 5 min in scanning mode using a Varioskan Flash Multi-Mode Microplate Reader (Thermo) and its associated software, and the initial velocity was determined from the linear portion, at approximately 2 min. The background rate of thiolysis in the absence of FadA was subtracted from the measured enzymatic rate.

### Acetyl-CoA level assay of peritoneal macrophages

For the measurement of cytosolic acetyl-CoA, primary peritoneal macrophages were stimulated with different strains or components for 4 h and were lysed with lysis buffer (1% Triton X-100, 20 mM Tris-HCl, pH 7.4, 150 mM NaCl) on ice for 10 min. The lysates were spun at 20,000× *g* for 10 min at 4 °C, the pellets were discarded, and the supernatants were used for acetyl-CoA measurement with an Acetyl-CoA Assay Kit (Sigma). Freshly isolated primary peritoneal macrophages were rested in RPMI-1640 medium with 2 mM sodium acetate (NaAc) for 24 h at 37 °C in an incubator, and the level of acetyl-CoA was measured.

### qRT-PCR analysis

RNA preparation and qPCR analysis were performed as described previously using gene-specific primers (Supplementary Table S4). Total RNA was extracted with 1 mL of Trizol reagent in accordance with the instructions of the manufacturer (ThermoFisher). One microgram of total RNA was applied for cDNA synthesis using the ReverTra Ace^®^ qPCR RT Kit (Toyobo). Real-time quantitative PCR was performed using the SYBR RT-PCR Kit (Toyobo) in an LC480 thermocycler (Roche, Indianapolis, IN). The 2^−ΔΔCt^ method was adopted to analyze the relative gene expression; the gene expression was normalized to the expression of *gapdh* for eukaryotic cells and 16sRNA for prokaryotic cells. Real-time qPCR data were collected from at least three independent experiments, with three technical replicates per experiment.

### Western blotting

Standard western blotting procedures were used. Cell extracts or culture filtrates (50 μg) of mycobacteria were denatured in 1× sodium dodecyl sulfate (SDS) protein sample buffer and separated on a 10% or 12% SDS-polyacrylamide gel. They were then transferred to nitrocellulose membranes. Next, the membrane was blocked, incubated with primary antibodies, and washed three times before incubation with secondary antibodies. The concentrations of the primary antibodies were 1:1000 for anti-FadA, anti-ESAT-6, and anti-SigA antibodies. Horseradish peroxidase-conjugated goat anti-rabbit polyclonal antibody was used as the secondary antibody at a 1:5000 dilution. After a final wash, analysis was conducted using an ECL reagent (Thermo). Densitometric analysis of band intensity was performed using Image J software.

### Mice and infection

Animal procedures were approved by the Animal Experiment Administration Committee of Shanghai Pulmonary Hospital (K18-033), and this study was carried out in strict accordance with the China National Research Council’s Guide for Care and Use of Laboratory Animals. All surgeries were performed under sodium pentobarbital anesthesia, and all efforts were made to minimize suffering. Female SPF C57BL/6 mice were purchased from Shanghai SLAC Laboratory Animal Co., Ltd. (Shanghai, China). Female C3HeB/FeJ mice were purchased from Jackson Laboratory (Bar Harbor, ME). Totally, 6–8-week-old female C57BL/6 mice were used for macrophage separation. For in vitro H37Rv infection, mouse macrophages were infected with a single cell suspension of bacteria at a MOI of 1. Female C3HeB/FeJ mice (6–8 weeks old) were infected by an aerosol method with approximately 200 CFU per mice of the indicated bacterial strains (using a Glas-Col inhalation exposure system (Glas-col, Terre Haute, IN)^62^ at an Animal Biosafety Level-3 (ABSL-3) Laboratory. At 1-week post-infection, mice received 0.3 mg of anti-IL-6 mAb (BioXcell) or isotype-matched control Ab (rat IgG1) intranasally once a week for up to 4 weeks. Mice were sacrificed 1 day or 4 weeks after infection. Tissues from the left lung from infected mice were harvested and homogenized in 1 mL of PBS. Homogenates in 10-fold serial dilutions were plated on 7H10 agar supplemented with 10% ADC enrichment and incubated at 37 °C. Colonies were counted after 4 weeks of incubation at 37 °C in 5% CO_2_. For histological analysis, half of each lung from infected mice for 4 weeks was fixed in 4% neutral-buffered paraformaldehyde solution for 24 h and then embedded in paraffin. A series of sections with a thickness of 4–7 μm were then cut and stained with H&E or Ziehl-Neelsen (acid-fast bacillus) stain, in accordance with standard protocols. Imaging was performed by microscopy (TCS CP5II; Leica, Germany). The pathology was evaluated by pathologists in a blinded manner. All mice were age-, weight-, and sex-matched in each experiment. The sample size was based on empirical data from pilot experiments. No additional randomization or blinding was used to allocate experimental groups.

### Generation of IL-6 knockout zebrafish using CRISPR/Cas9 mutagenesis

To study the function of IL-6 in granuloma development, we employed the Cas9/gRNA system to generate IL-6 knockout zebrafish. *il6*^12d2i^ and *il6*^20dl^ mutant zebrafish were constructed at the China Zebrafish Resource Center (CZRC), as described previously^63^. In brief, a functional gRNA targeting the second exon of the *il6* gene was designed and microinjected with Cas9 mRNA into one-cell embryos of zebrafish. The F_0_-generation fish grew to adulthood. Individual outcrosses of the F_0_ -zebrafish with the WT AB fish allowed us to screen for germline-transmitted mutations and to identify mutations of interest in the F_1_-progeny. The F_1_-progeny were screened from the sequencing results of the tailfin DNA from the adult zebrafish. The following primers were used for sequencing: (F) 5′-CAGTGCTATTCCTGTCTGCTAC-3′ and (R) 5′-TAACTGGGTTACTCGTTTTGAGT-3′. The F_1_-zebrafish carrying individual mutations of interest were spawned together to obtain F_2_-generation progeny for the experiments. Finally, a total of two zebrafish lines bearing different *il6* mutations resulting in premature stop codons after 65 and 70 amino acids, were used in the study. These two different *il6* null mutant zebrafish lines were named *il6*^12d2i^ (−10 bp, loss of CTGTACAAGGAC and insert of GA) and *il6*^20dl^ (−20 bp, loss of CTGTACAAGGACGTGAAGAC). qPCR was used to confirm the deletion of *il6*.

### Zebrafish infection

WT zebrafish (*Danio rerio*) were obtained from CZRC (Wuhan) and husbandry was performed as described previously by Swaim et al.^64^. Briefly, the fish were reared in recirculating fish systems obtained from Qingdao Elvin Marine Technology Co., Ltd. (Qingdao, China), and transferred to a flowthrough fish system for the infection experiment. Up to 10 fish were kept in a 3 L tank and tanks were maintained under standard conditions for housing zebrafish (water temperature ~28 °C, pH ~7.4, and conductivity ~1500 μS). Infection protocols approved by the Animal Experiment Administration Committee were used for the zebrafish infection studies. Healthy adult fish were infected as described previously^64^. Fish were infected by intraperitoneal injection with approximately 200 CFU of the indicated bacterial strains after first anesthetizing with 0.1% 3-aminobenzoic acid ethyl ester (tricaine; Sigma). Bacterial CFU contained in the injected inoculum was confirmed by plating onto 7H10 agar. To assess the bacterial burden from whole fish post-infection, the fish infected for 1 or 14 days were terminally anesthetized in 0.5% tricaine, homogenized in 1.0 mL of PBS, and then the dilutions were plated on 7H10 agar plates. Three or five fish were plated for each condition and the resulting bacterial counts were determined. For histological analysis, fish infected for 14 days were fixed in 4% neutral-buffered paraformaldehyde solution for 72 h, embedded in paraffin, sectioned, and stained with H&E or Ziehl–Neelsen stain. According to the methods described previously^15,65^, acid-fast staining of granulomas demonstrates the presence of acid-fast bacilli and bacterial burden in the granulomas, H&E staining indicates the necrotic regions of granulomas. Granulomas of different *M. marinum*-infected adult zebrafish scored for *M. marinum* burden as less than 10 or 10 or more bacteria and the percentage of necrotic granulomas was quantified and compared based on the staining results. A total number of granulomas for each strain was counted and shown with “*n*”, and the different number of granulomas between different groups also reflected the pathology of *M. marinum*-infected zebrafish.

### Statistical analysis

Data from independent experiments were expressed as the mean ± SEM. All experimental data were analyzed using GraphPad Prism software. The statistical significance of differences between the two groups was determined by a two-tailed unpaired Student’s *t-*test. One-way or two-way ANOVA with Bonferroni’s multiple comparisons test was used for statistical analysis when comparing more than two groups. Populations were compared by Fisher’s exact test. Differences were considered significant at *P* < 0.05.

## Supplementary information

Supplementary Information
