# Supplementary material for: Interception of host fatty acid metabolism by mycobacteria under hypoxia to suppress anti-TB immunity
Source: Cell Discov. 2021 Oct 5;7:90. doi: 10.1038/s41421-021-00301-1 (PMC8490369; doi:10.1038/s41421-021-00301-1)
Supplement: Supplementary file 1 — Supplementary Information [file 41421_2021_301_MOESM1_ESM.pdf]

Supplementary Information for

Interception of host fatty acid metabolism by mycobacteria under hypoxia to suppress anti-TB immunity

Hua Yang[1#], Fei Wang[2#], Xinya Guo[1#], Feng Liu[1#], Zhonghua Liu[1], Xiangyang Wu[2], Mengmeng Zhao[2], Mingtong Ma[2], Haipeng Liu[1], Lianhua Qin[1], Lin Wang[1], Tianqi Tang[2], Wei Sha[3], Yang Wang[2], Jianxia Chen[1], Xiaochen Huang[1], Jie Wang[1], Cheng Peng[2], Ruijuan Zheng[1], Fen Tang[2], Lu Zhang[4], Chunyan Wu[5], Stefan H Oehlers[6], Zhigang Song[7], Jialei She[7], Hua Feng[8], Xunwei Xie[9], Baoxue Ge[1,2*]

Correspondence: gebaoxue@sibs.ac.cn

This PDF file includes:

Figures S1 to S12
Tables S1 to S4

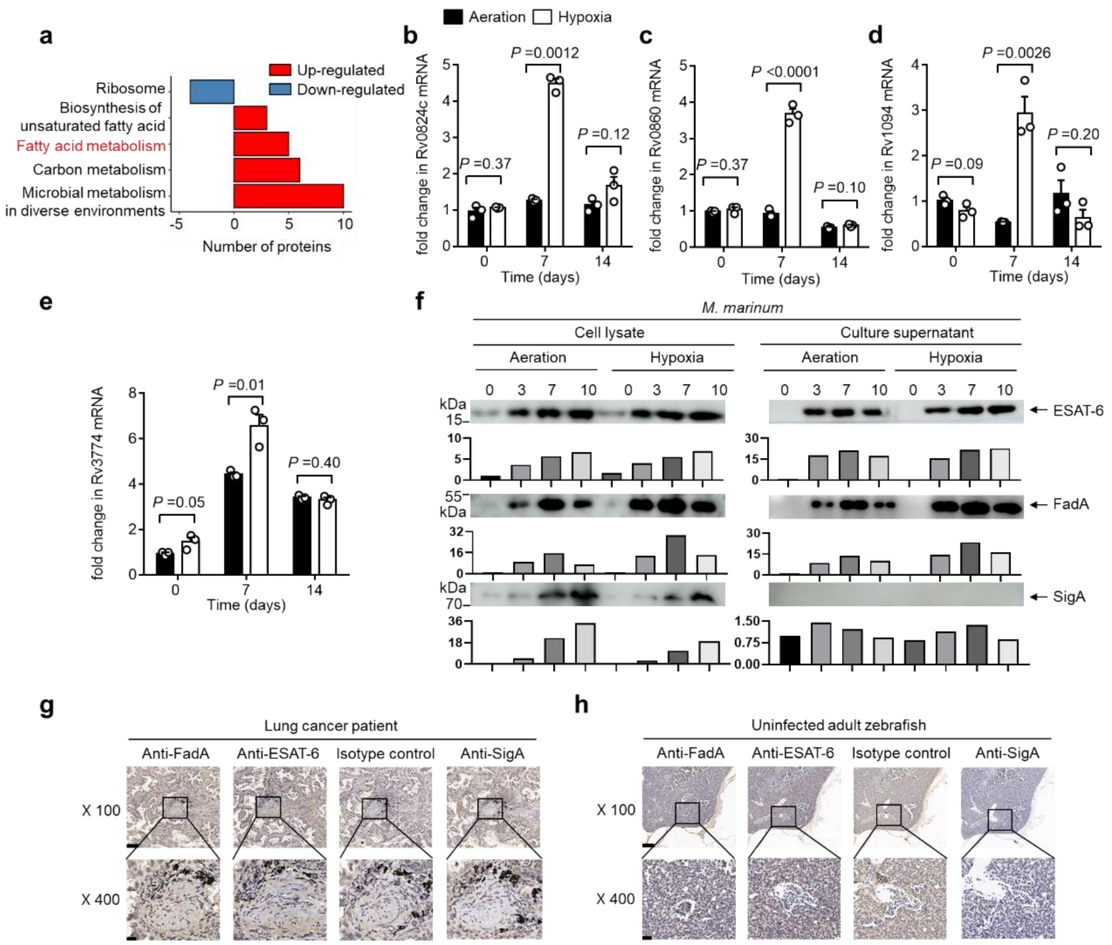

Supplementary Fig. S1. Hypoxia induces FadA. a Kyoto Encyclopedia of Genes and Genomes (KEGG) pathway enrichment of differentially secreted proteins by comparing hypoxia with aeration. **b-e** Quantitative polymerase chain reaction (qPCR) analysis of Rv0824c (**b**), Rv0860 (**c**), Rv1094 (**d**), Rv3774 (**e**) mRNA from H37Rv incubated under aeration and hypoxia for 0, 7 or 14 days *in vitro* (mean ± s.e.m.). Data are representative of one experiment with at least three independent biological replicates; each circle represents one technical repeat. Bar charts show means. **f** Immunoblot (IB) of cell lysate and culture filtrate from *M. marinum* incubated under aeration and hypoxia for 0, 3, 7 or 10 days with anti-FadA, anti-ESAT-6 and anti-SigA antibodies at a 1:1000 dilution. Bars represent densitometric analysis of band intensity. **g, h** Immunolocalization of FadA in lung granuloma sections from lung cancer patient (**g**) and whole fish sections of uninfected adult zebrafish (**h**) with anti-FadA polyclonal antibody at a 1:100 dilution and anti-rabbit secondary antibody labeled with HRP at a 1:200 dilution (scale bar, 100 μm (top) and 20 μm (bottom)), compared with anti-ESAT-6 polyclonal antibody at a 1:200 dilution, isotype polyclonal control antibody at a 1:100 dilution and anti-SigA antibody labeled with HRP at 1:100 dilution (scale bar, 100 μm

(top) and 20 μm (bottom)). Results in **f-h** are representative of three independent experiments. Two-tailed unpaired Student's *t*-test (**b-e**) was used for statistical analysis.

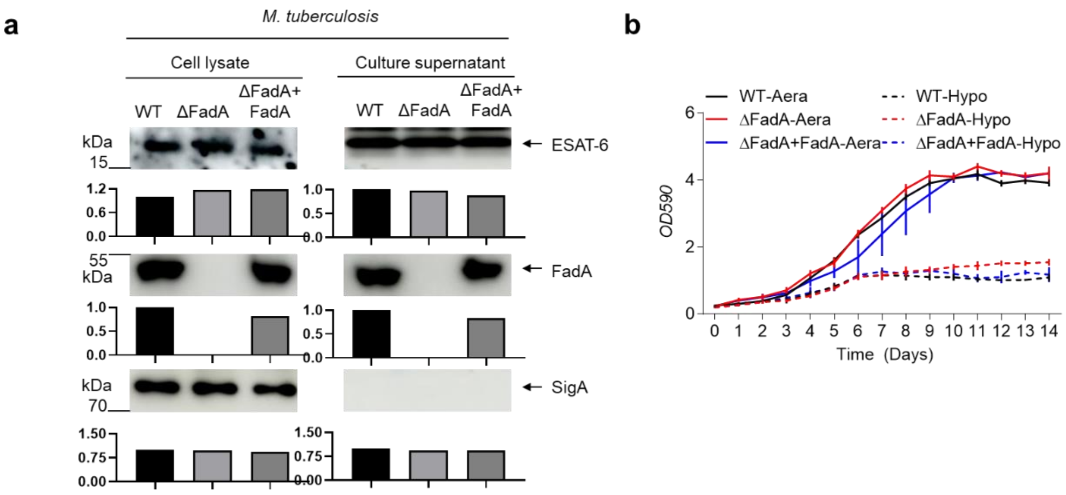

Supplementary Fig. S2. IB and Growth curve *in vitro* of H37Rv, H37RvΔFadA or H37Rv(ΔFadA+FadA) strains. a IB of cell lysate and culture filtrate of H37Rv, H37RvΔFadA or H37Rv(ΔFadA+FadA) strains with anti-FadA, anti-ESAT-6 and anti-SigA antibodies at a 1:1000 dilution. Bars represent densitometric analysis of band intensity. Results are representative of three independent experiments. **b** Growth curve *in vitro* of H37Rv, H37RvΔFadA or H37Rv(ΔFadA+FadA) strains under aeration or hypoxia. The strains were grown to mid-log phase and the growth curve was measured using a Bioscreen Growth Curve Instrument. Hypoxic conditions were established by covering bacterial suspensions with paraffin oil. The optical density was measured at an absorbance of 590 nm every day. Cultures of H37Rv, H37RvΔFadA or H37Rv(ΔFadA+FadA) strains were grown at 37 °C for 14 days. Data are representative of one experiment with at least three independent biological replicates.

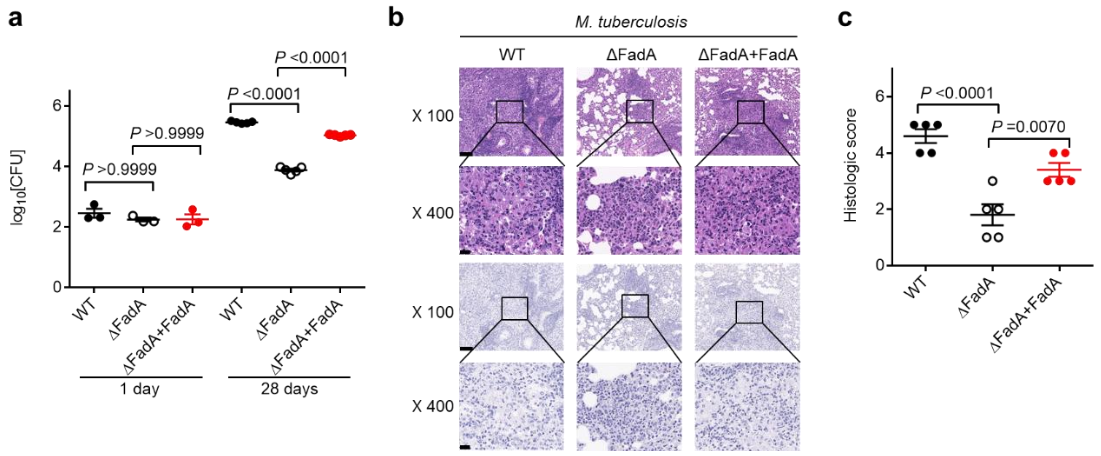

Supplementary Fig. S3. Pathology assessment in lung sections of C3HeB/FeJ mice infected with H37Rv, H37RvΔFadA or H37Rv(ΔFadA+FadA) strains. a-c Six-week-old female C3HeB/FeJ mice were aerosol-infected with roughly 200 colony-forming unit (CFU) per mouse of H37Rv, H37RvΔFadA or H37Rv(ΔFadA+FadA) strains for 1 day or 4 weeks. Histopathology was assessed in lung sections by bacterial CFU counting (**a**; mean ± s.e.m. of n=3 mice infected for 1 day or n=5 mice infected for 4 weeks), haematoxylin and eosin (H&E) and acid-fast staining from lungs of mice infected for 4 weeks (**b**; representative of one experiment with at least three independent replicates; scale bar, 100 μm (top) and 20 μm (bottom)) and histologic score (**c**). One-way ANOVA with Bonferroni's multiple comparisons test (**a, c**) was used for statistical analysis.

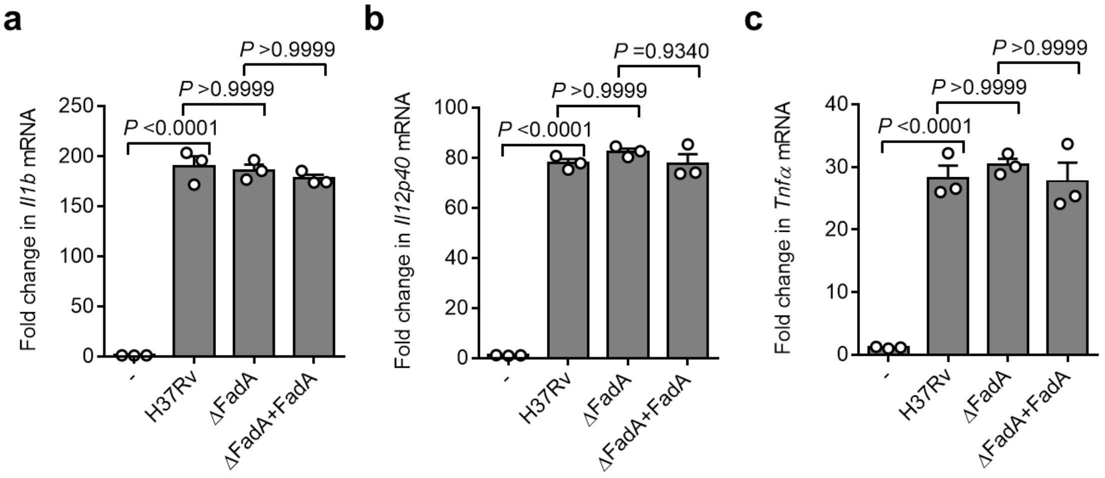

Supplementary Fig. S4. qPCR analysis of *Il1b* (**a**), *Il12 p40* (**b**) and *Tnfα* (**c**) mRNA from peritoneal macrophages infected with H37Rv, H37RvΔFadA or H37Rv(ΔFadA+FadA) strains for 4h (MOI=1) (mean ± s.e.m.). Data are representative of one experiment with at least three independent biological replicates; each circle represents one technical repeat. Bar charts show means. One-way ANOVA with Bonferroni's multiple comparisons test (**a-c**) was used for statistical analysis.

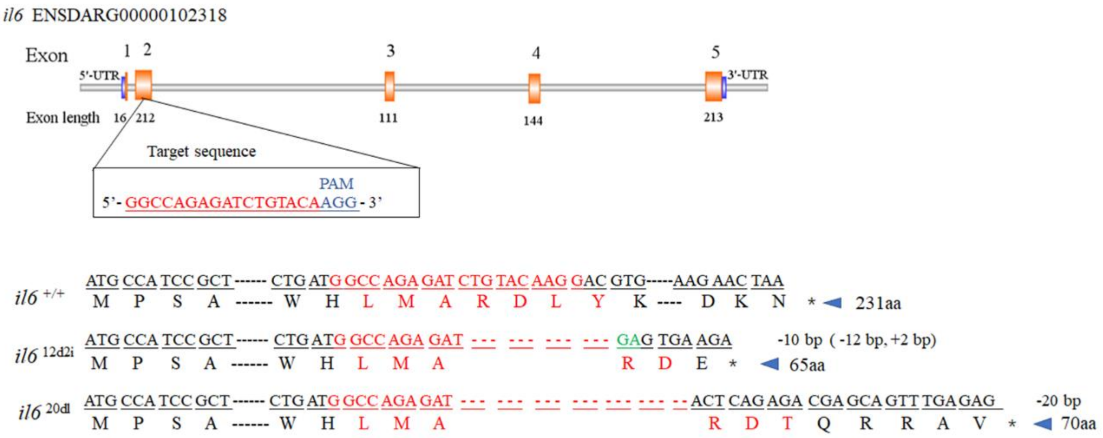

Supplementary Fig. S5. Generation of *il6* ^{12d2i} and *il6* ^{20dl} mutant zebrafish lines using CRISPR-Cas9 mutagenesis. The Cas9/gRNA system was employed to generate IL-6 knockout zebrafish, which was constructed by the China Zebrafish Resource Center (CZRC) as described previously. An appropriate guide RNA (gRNA) target site was identified in the second exon of *il6*. gRNA target sites were sequenced from F_1 -generation mutant zebrafish and two frameshift mutations (*il6* ^{12d2i}: −10 bp deletion and +2 bp insertion; and *il6* ^{20dl}: -20bp deletion) detected, leading to truncated protein products of 65 and 70 amino acids, respectively.

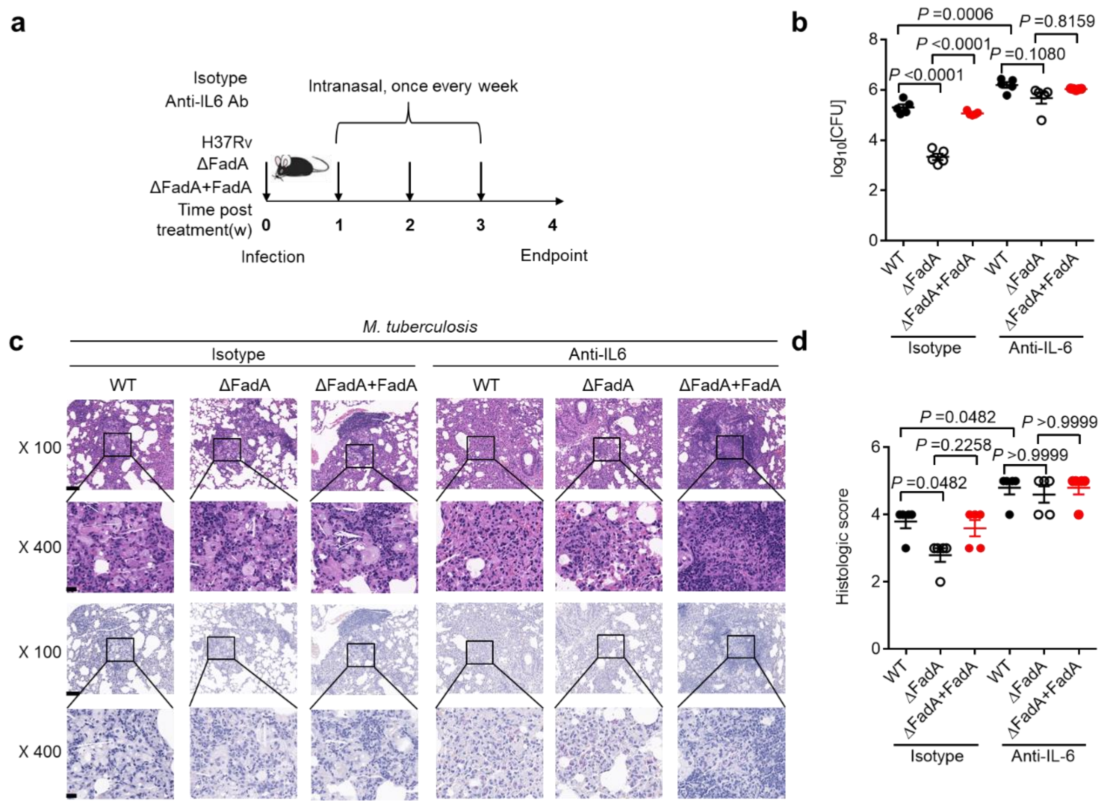

Supplementary Fig. S6. Pathology assessment in lung sections of C3HeB/FeJ mice infected with H37Rv, H37RvΔFadA or H37Rv(ΔFadA+FadA) **strains treated with neutralizing anti-IL-6 mAb. a** Diagram showing the procedure for H37Rv, H37RvΔFadA or H37Rv(ΔFadA+FadA) strains infected C3HeB/FeJ mice treated with a neutralizing anti-IL-6 or an isotype-matched control mAb. **b-d** Six-week-old female C3HeB/FeJ mice were aerosol-infected with roughly 200 CFU/mouse of H37Rv, H37RvΔFadA or H37Rv(ΔFadA+FadA) strains. At 1-week post infection, mice received 0.3 mg of anti-IL-6 mAb (BioXcell) or isotype-matched control Ab (rat IgG1) intranasally once 1 week for up to 4 weeks. Histopathology was assessed in lung sections by bacterial CFU counting (**b**; mean ± s.e.m. of n=5 mice infected for 4 weeks), H&E and acid-fast staining from lungs of mice infected for 4 weeks (**c**; representative of one experiment with at least three independent replicates; scale bar, 100 μm (top) and 20 μm (bottom)) and histologic score (**d**). One-way ANOVA with Bonferroni's multiple comparisons test (**b, d**) was used for statistical analysis.

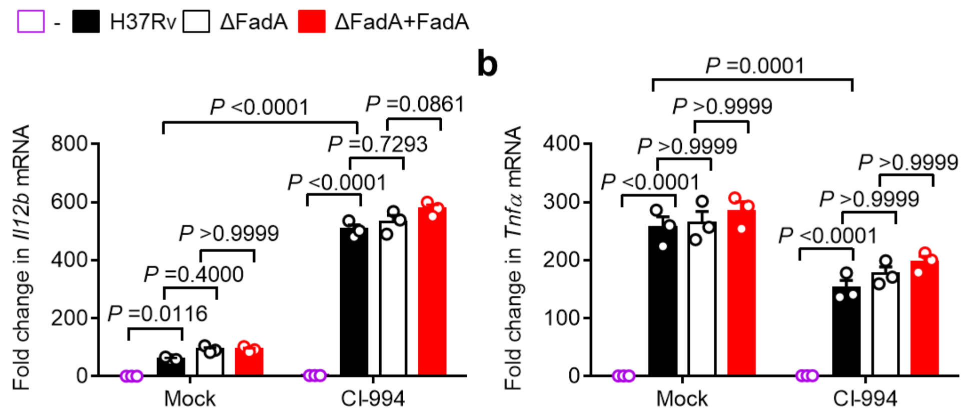

Supplementary Fig. S7. FadA suppresses Il-6 through H3K9Ac. a, b qPCR analysis of *Il12b*(**a**) or *Tnfα*(**b**) mRNA from control or histone deacetylase (HDAC) 1-3 inhibitor CI-994-pretreated peritoneal macrophages infected with H37Rv, H37RvΔFadA or H37Rv(ΔFadA+FadA) strains for 4h (MOI=1) (mean ± s.e.m.). Data in **a-b** represent one experiment with at least three independent replicates. Two-way ANOVA with Bonferroni's multiple comparisons test (**a-b**) was used for statistical analysis (ns, not significant; **** $P< 0.0001$).

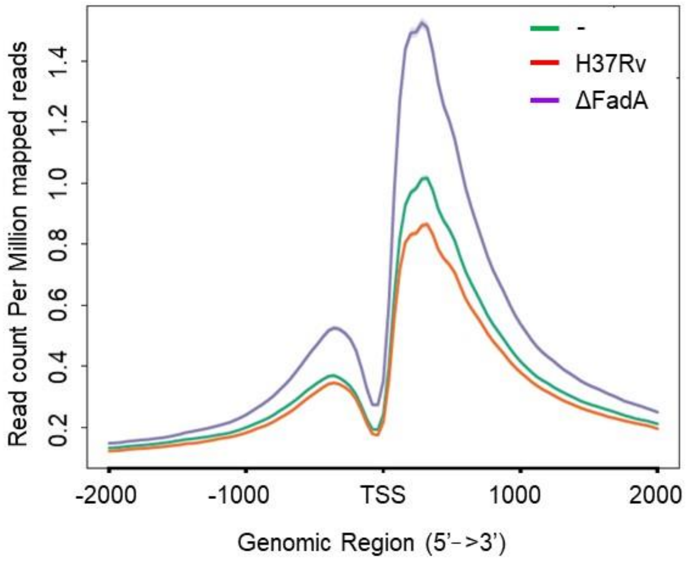

Supplementary Fig. S8. FadA downregulates the general H3K9Ac enrichment. ChIP-seq analysis of histone H3 acetylation at the lysine 9 residue (H3K9Ac) of peritoneal macrophage infected with H37Rv or H37RvΔFadA strains (MOI=1) for 4h with SimpleChIP Enzymatic Chromatin IP Kit and the Illumina Hiseq Xten platforms at the CAS-MPG Partner Institute for Computational Biology Omics Core.

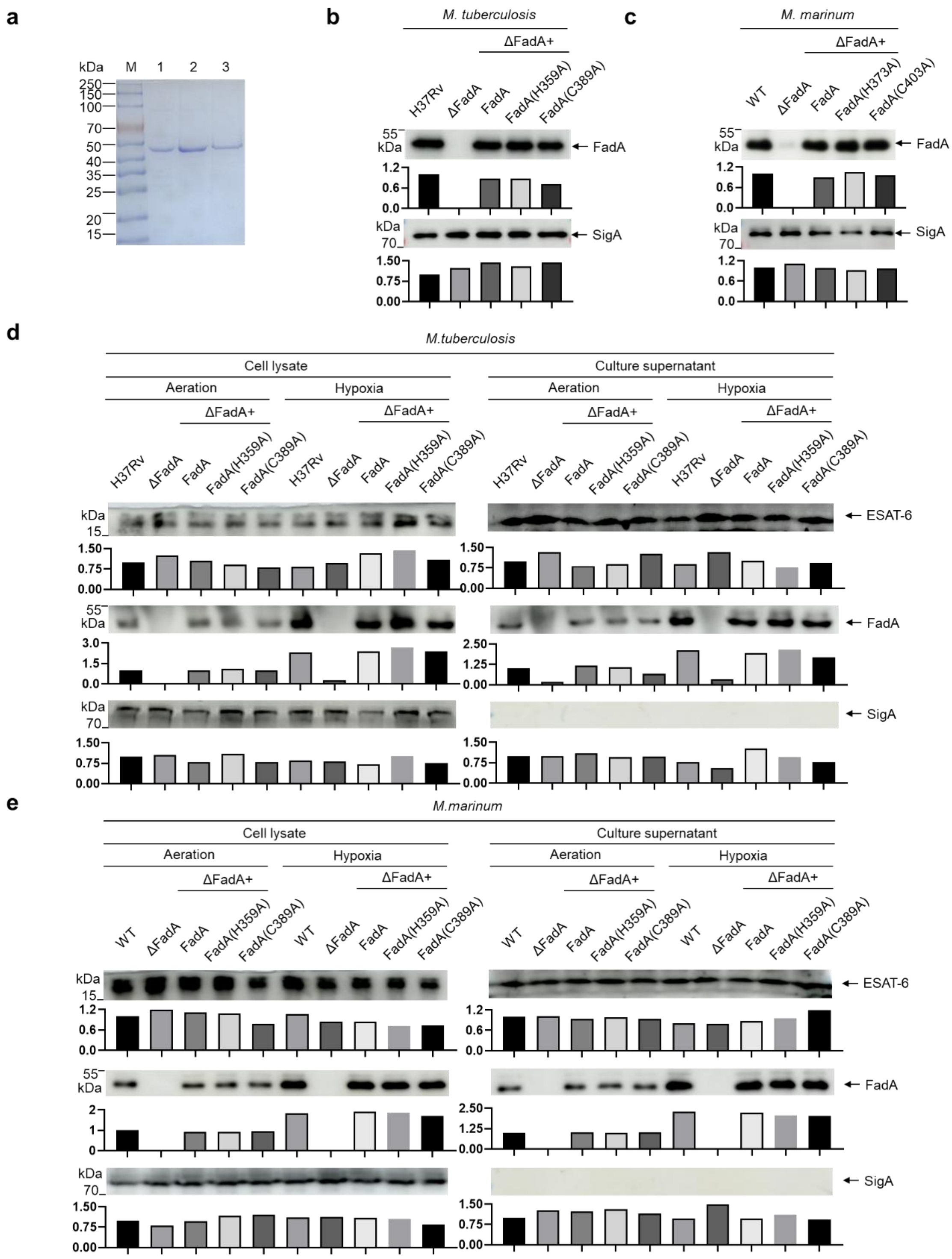

Supplementary Fig. S9. IB of various FadA mutants in *M. tuberculosis* and *M. marinum* strains. a Analysis of the purified FadA recombinant protein and its derivative mutant protein. Construction and purification of FadA recombinant protein and its derivative mutants were performed as described before and analyzed by Dodecyl sulfate, sodium salt -Polyacrylamide gel electrophoresis (SDS-PAGE). M. PageRuler

prestained protein ladder (Fermentas); lane 1. the purified FadA protein with an expected molecular weight of 43.97 kDa; lane 2. the purified FadA(H359A) protein with an expected molecular weight of 43.90 kDa; lane 3. the purified FadA(C389A) protein with an expected molecular weight of 43.94 kDa. **b** IB of lysate of H37Rv, H37RvΔFadA, H37Rv(ΔFadA+FadA), H37Rv(ΔFadA+FadA(H359A)), and H37Rv(ΔFadA+FadA(C389A)) strains with anti-FadA antibody and control anti-SigA antibody at a 1:1000 dilution. **c** IB of lysate of WT, ΔFadA, ΔFadA+FadA, ΔFadA+FadA(H373A), and ΔFadA+FadA(C403A) *M. marinum* strains with anti-FadA antibody and control anti-SigA antibody at a 1:1000 dilution. **d** IB of culture filtrate and cell lysate from H37Rv, H37RvΔFadA, H37Rv(ΔFadA+FadA), H37Rv(ΔFadA+FadA(H359A)), and H37Rv(ΔFadA+FadA(C389A)) strains incubated under aeration and hypoxia for 7 days with anti-FadA, anti-ESAT-6 and anti-SigA antibodies at a 1:1000 dilution. **e** IB of culture filtrate and cell lysate from WT, ΔFadA, ΔFadA+FadA, ΔFadA+FadA(H373A), and ΔFadA+FadA(C403A) *M. marinum* strains incubated under aeration and hypoxia for 7 days with anti-FadA, anti-ESAT-6 and anti-SigA antibodies at a 1:1000 dilution. Bars represent densitometric analysis of band intensity. Results are representative of three independent experiments.

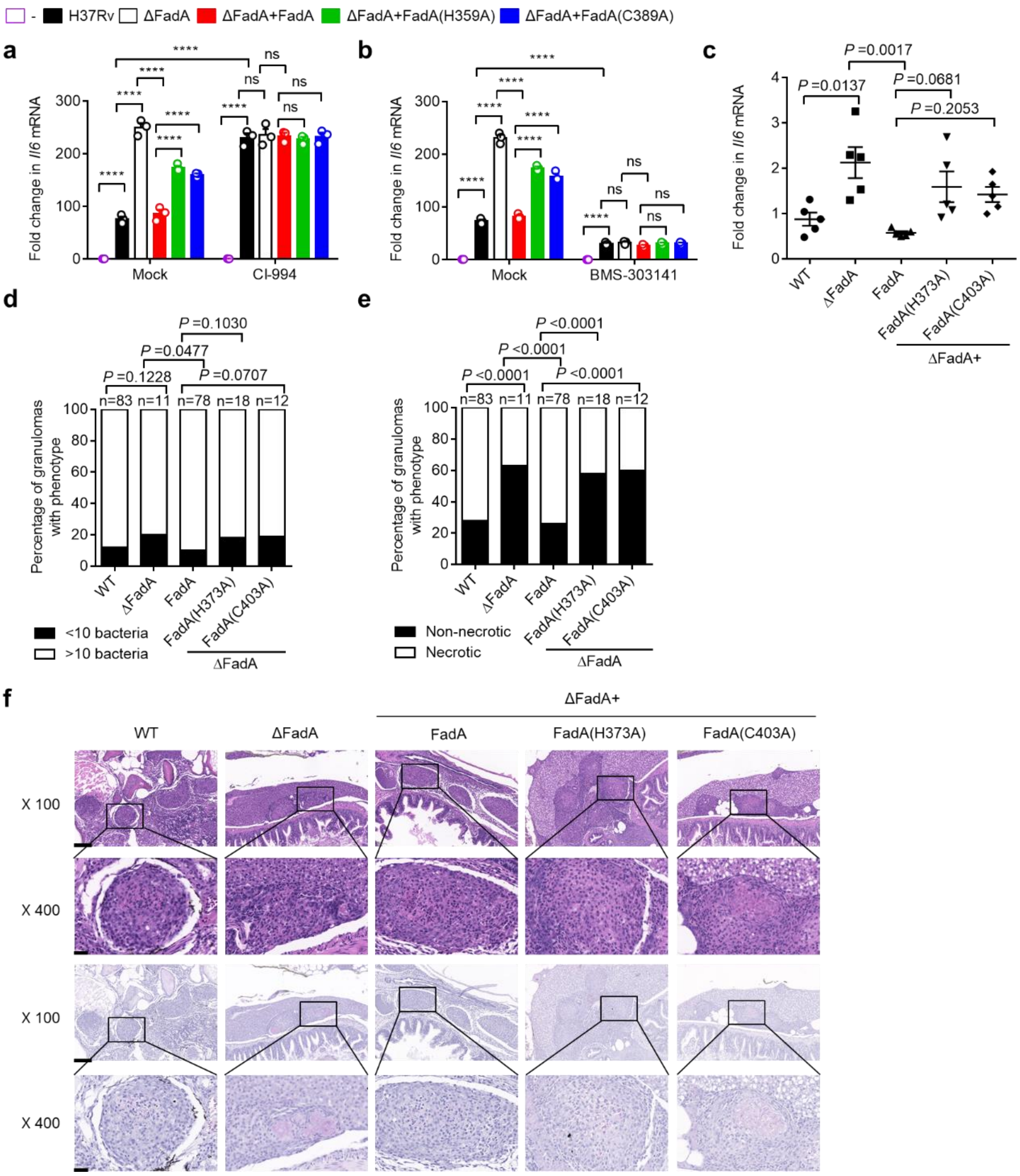

Supplementary Fig. S10. FadA functions through its acetyltransferase activity. a qPCR analysis of *Il6* mRNA from control or HDAC 1-3 inhibitor CI-994-pretreated peritoneal macrophages infected with H37Rv, H37RvΔFadA, H37Rv(ΔFadA+FadA), H37Rv(ΔFadA+FadA(H359A)), and H37Rv(ΔFadA+FadA(C389A)) strains for 4h (MOI=1) (mean ± s.e.m.). **b** qPCR analysis of *Il6* mRNA from control or ATP-citrate lyase (ACL) inhibitor BMS-303141-pretreated peritoneal macrophages infected with H37Rv, H37RvΔFadA, H37Rv(ΔFadA+FadA), H37Rv(ΔFadA+FadA(H359A)), and H37Rv(ΔFadA+FadA(C389A)) strains for 4h (MOI=1) (mean ± s.e.m.). Data in **a, b** are representative of one experiment with at least three independent biological replicates; each circle represents one technical repeat. **c** qPCR analysis of *Il6* mRNA

from adult zebrafish intraperitoneally infected with roughly 200 CFU of WT, ΔFadA, ΔFadA+FadA, ΔFadA+FadA(H373A), ΔFadA+FadA(C403A) *M. marinum* strains for 14 days (mean ± s.e.m. of n=5). **d-f** Adult zebrafish were intraperitoneally infected with roughly 200 CFUs of WT, ΔFadA, ΔFadA+FadA, ΔFadA+FadA(H373A), and ΔFadA+FadA(C403A) *M. marinum* strains for 14 days. Histopathology was assessed in the whole fish bycomparison of granulomas between different *M. marinum*-infected adult zebrafish scored for *M. marinum* burden as less than 10 or 10 or more bacteria (**d**), or percentage of necrotic granulomas in each fish (**e**) and H&E or acid-fast staining (**f;** representative of one experiment with at least three independent replicates; scale bar, 100 μm (top) and 20 μm (bottom)). "n" in **g, h** was the total number of granulomas for each strain infected fish. Total number of zebrafish analyzed: 5 (WT), 5 (ΔFadA), 5 (ΔFadA+FadA), 5 (ΔFadA+FadA(H373A)), 5 (ΔFadA+ FadA(C403A)). Data in **c-f** represent one experiment with at least three independent replicates. One-way (**c**) or two-way (**a, b**) ANOVA with Bonferroni's multiple comparisons test and Fisher's exact test (**d, e**) were used for statistical analysis (ns, not significant; **** $P < 0.0001$).

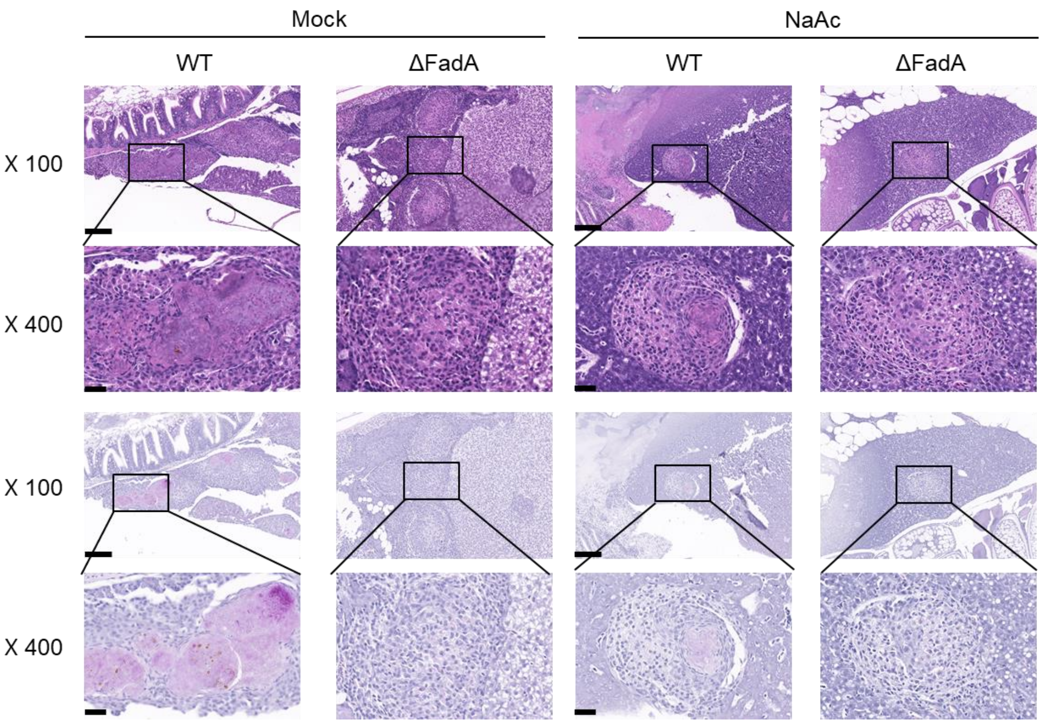

Supplementary Fig. S11. Pathology assessment in adult zebrafish infected with *M. marinum* strains treated with sodium acetate (NaAc). Adult zebrafish were intraperitoneally infected with roughly 200 CFU of WT or ΔFadA *M. marinum* strains for 14 days. NaAc was injected intraperitoneally to a final concentration of 2 mM immediately after infection and reinjected every 3 days. Histopathology was assessed in the whole fish by H&E or acid-fast staining (representative of one experiment with at least three independent replicates; scale bar, 100 µm (top) and 20 µm (bottom)).

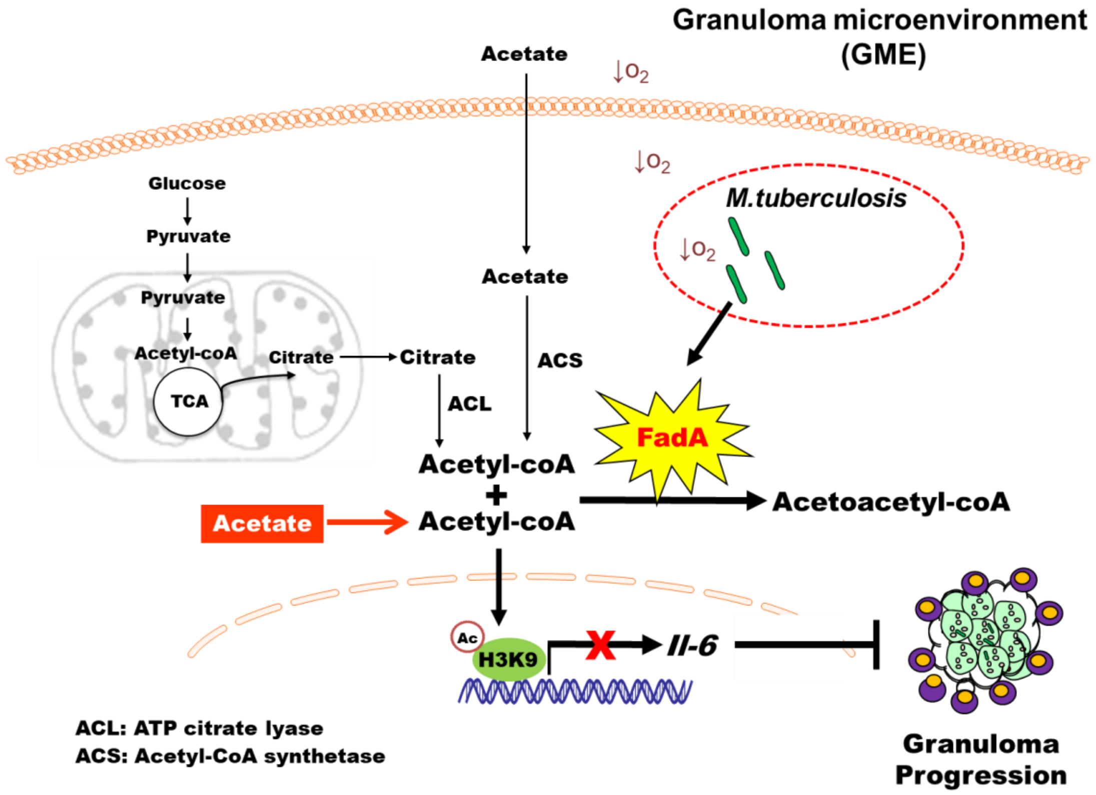

Supplementary Fig. S12. An interception model to host fatty acid metabolism by mycobacteria under hypoxia to suppress anti-TB immunity. *M. tuberculosis* utilizes numerous strategies for immune evasion**.** Intriguingly, mycobacteria under hypoxia markedly secrete the protein FadA, which acts as an acetyltransferase that converts host acetyl-CoA to acetoacetyl-CoA to reduce the host acetyl-CoA level and then suppress the histone H3K9 acetylation-mediated expression of *Il6*, thus promoting the progression of granuloma. Moreover, supplementation of acetate increases the acetyl-CoA level and inhibits the enhancement effect of FadA on the mycobacterial growth and the progression of granuloma.

Supplementary Table S1. Upregulated secreted proteins of H37Rv under hypoxia

Gene ID	Protein	Protein description	Ratio (Hypo/Aero)	*p*-value
Rv2059	O07257	Zinc/manganese transporter substrate-binding protein	1.855	0.00218
Rv0824	I6XWD0	Acyl-[acyl-carrier-protein] desaturase	1.724	0.00001
Rv2031c	I6Y869	Heat shock protein hspX	1.723	0.00000
Rv0467	I6Y7W3	Isocitrate lyase	1.661	0.00000
Rv3418	I6Y3F9	10 kDa chaperonin	1.618	0.00000
Rv1133c	I6Y5P6	5-methyltetrahydropteroyltriglutamate--homocysteine methyltransferase	1.600	0.00000
Rv1837c	I6X2F8	Malate synthase G	1.582	0.00011
Rv0860	I6Y8Y4	3-hydroxyacyl-CoA dehydrogenase/enoyl-CoA hydratase/3-hydroxybutyryl-CoA epimerase	1.576	0.00000
Rv0859	I6X9Z1	Acetyl-CoA C-acetyltransferase	1.565	0.01039
Rv0670	I6Y4E3	Probable endonuclease 4	1.526	0.00162
Rv3042	I6X638	Phosphoserine phosphatase	1.512	0.04787
Rv1094	I6Y5K6	Acyl-[acyl-carrier-protein] desaturase	1.497	0.00000
Rv2391	I6YDA2	Sulfite reductase [ferredoxin]	1.464	0.04162
Rv0251c	O53673	Heat shock protein hsp	1.457	0.03051
Rv2996	I6X5Z2	D-3-phosphoglycerate dehydrogenase	1.445	0.00000
Rv2711	I6XF43	Iron-dependent repressor IdeR	1.440	0.00551

Rv3774	P75019	Enoyl-CoA hydratase	1.387	0.00081
Rv3841	I6YD59	Bacterioferritin bfrB	1.368	0.00000
Rv0815c	I6X9V7	Sulfurtransferase	1.366	0.00000
Rv2626c	I6Y193	Hypoxic response protein 1	1.332	0.00390
Rv0211	I6Y334	Phosphoenolpyruvate carboxykinase [GTP]	1.324	0.00851
Rv0475	I6X969	Heparin-binding hemagglutinin	1.311	0.00002

Supplementary Table S2. Downregulated secreted proteins of H37Rv under hypoxia

Gene ID	Protein	Protein description	Ratio (Hypo/Aero)	*p*-value
Rv2346c	I6XE37	ESAT-6-like protein esxN	0.244	0.02910
Rv1793	I6Y7I0	ESAT-6-like protein esxN	0.253	0.03389
Rv3616c	I6XHT2	ESX-1 secretion-associated protein EspA	0.274	0.01934
Rv3615c	I6Y444	ESX-1 secretion-associated protein EspC	0.322	0.01972
Rv0379	I6X919	Preprotein translocase subunit secE2	0.391	0.00126
Rv1197	I6X0L2	ESAT-6-like protein esxK	0.396	0.00002
Rv1813c	I6Y7J9	Uncharacterized protein	0.419	0.03948
Rv2226	I6X3M7	Uncharacterized protein	0.534	0.01440
Rv3801c	I6YD25	Fatty acid CoA ligase FadD32	0.554	0.01320
Rv3874	B5TV88	10 kDa culture filtrate protein	0.574	0.00035
Rv0088	I6X8H2	Uncharacterized protein	0.590	0.00646
Rv0707	I6Y8I3	30S ribosomal protein S3	0.609	0.02991
Rv3800c	I6X8D2	Polyketide synthase 13	0.613	0.00000
Rv0721	I6X9M1	30S ribosomal protein S5	0.630	0.00024
Rv0685	I6Y4F5	Elongation factor Tu	0.643	0.00002
Rv1392	I6X149	S-adenosylmethionine synthase	0.656	0.03341
Rv0928	I6XWK9	Phosphate-binding protein PstS	0.657	0.00151

Rv2984	A5YKM2	Polyphosphate kinase	0.662	0.02981
Rv3597c	I6X7R6	Protein lsr2	0.671	0.00540
Rv2909c	I6X5P6	30S ribosomal protein S16	0.685	0.01179
Rv3596c	I6YGL7	ATP-dependent Clp protease ATP-binding subunit ClpC	0.695	0.02442
Rv2215	I6XDT2	2-oxoglutarate dehydrogenase, E2 component, dihydrolipoamide succinyltransferase	0.708	0.00048
Rv2908c	I6YEP3	UPF0109 protein RVBD_2908c	0.713	0.00617
Rv3458c	I6X7D0	30S ribosomal protein S4	0.722	0.02838
Rv3859c	I6Y4Q0	Glutamate synthase (NADPH/NADH) large chain	0.729	0.03078
Rv1876	I6YBU4	Bacterioferritin	0.732	0.01145
Rv0066c	O53611	Isocitrate dehydrogenase, NADP-dependent	0.733	0.00066
Rv0667	I6XVX4	DNA-directed RNA polymerase subunit beta	0.748	0.00013
Rv1060	O53408	Uncharacterized protein	0.761	0.03443

Supplementary Table S3 Bacterial strains and plasmids used in this study.

Name	Description	Reference
**Strains**		
E. coli DH5α	F- 80*lac*Z M15 (lacZYA–argF)U169*eoR recA1 endA1 hsdR17 phoA* *supE*44-thi-1 *gyrA96 relA1*	Laboratory stock
E. coli BL21	F- *ompT gal dcm lon hsdSB*(rB- mB-) λ(DE3 [*lacI lacUV*5-T7 gene 1 *ind1 sam7 nin5*])	Laboratory stock
M. smegmatis mc^{2}*155*	wild type *M. smegmatis* reference strain	Laboratory stock
M. tuberculosis H37Rv	wild type *M. tuberculosis* virulent reference strain	Laboratory stock
M. tuberculosis H37Ra	wild type *M. tuberculosis* avirulent reference strain	Laboratory stock
M. marinum Aronson (BAA-535)	wild type *M. marinum* reference strain	Gift from Dr. Zhang L
H37RvΔFadA	*M. tuberculosis* H_{37}Rv Δ FadA ::hyg	This study

H37Rv(ΔFadA+FadA)	*M. tuberculosis* $H_{37}Rv$ ΔFadA+FadA	This study
H37Rv(ΔFadA+FadA(H359A))	*M. tuberculosis* $H_{37}Rv$ ΔFadA+FadA (H359A)	This study
H37Rv(ΔFadA+FadA(C389A))	*M. tuberculosis* $H_{37}Rv$ ΔFadA+FadA (C389A)	This study
ΔFadA (*M. marinum*)	*M. marinum* Aronson ΔFadA ::hyg	This study
ΔFadA +FadA (*M. marinum*)	*M. marinum* Aronson ΔFadA +FadA	This study
ΔFadA +FadA(H373A) (*M. marinum*)	*M. marinum* Aronson ΔFadA +FadA (H373A)	This study
ΔFadA +FadA(C403A) (*M. marinum*)	*M. marinum* Aronson ΔFadA +FadA (C403A)	This study
**Vector Plasmid**		
pET28a	Expression vector	Laboratory stock
pVV16	*E. coli*/ Mycobacterium shuttle plasmid	Laboratory stock
pYUB854	Cosmid vector containing allelic exchange substrates	Gift from Dr. Lyu LD

phAE87	Temperature-sensitive shuttle phasmid to generate a specialized transducing mycobacteriophage	Gift from Dr. Lyu LD
pET28a-FadA	Kan^R, pET28a harboring Rv0859	This study
pET28a-FadA(H359A)	Kan^R, pET28a harboring Rv0859(H359A)	This study
pET28a-FadA(C389A)	Kan^R, pET28a harboring Rv0859(C389A)	This study
pVV16-Rv0859	Kan^R, Hyg^R, pVV16 harboring Rv0859	This study
pVV16-Rv0859(H359A)	Kan^R, Hyg^R, pVV16 harboring Rv0859(H359A)	This study
pVV16-Rv0859(C389A)	Kan^R, Hyg^R, pVV16 harboring Rv0859(C389A)	This study
pVV16- MMAR_4677	Kan^R, Hyg^R, pVV16 harboring MMAR_4677	This study
pVV16-MMAR_4677(H373A)	Kan^R, Hyg^R, pVV16 harboring MMAR_4677(H373A)	This study
pVV16- MMAR_4677(C403A)	Kan^R, Hyg^R, pVV16 harboring MMAR_4677(C403A)	This study

Supplementary Table S4 Primers used in this study.

Primer name	Sequence (5'-3')
mIL-1β-F	AGAAACAGTCCAGCCCATAC
mIL-1β-R	CTGGTACATCAGCACCTCAC
mIL-6-F	TCCAGTTGCCTTCTTGGGAC
mIL-6-R	GTGTAATTAAGCCTCCGACTTG
mIL-12P40-F	GAGCACTCCCCATTCCTACT
mIL-12P40-R	CCCTCCTCTGTCTCCTTCAT
mTNF-α-F	TTCTGTCTACTGAACTTCGGGGTGATCGGTCC
mTNF-α-R	GTATGAGATAGCAAATCGGCTGACGGTGTGGG
mGAPDH-F	CCCACTAACATCAAATGGGG
mGAPDH-R	CCTTCCACAATGCCAAAGTT
pVV16-Rv0859-F	GGAATCACTTCCATatgtccgaagaagcc
pVV16-Rv0859-R	GTGGTGGTGAAGCTTaaccctctcgatgatc
pVV16-Mm4677-F	GGAATCACTTCCATatgacggatctgaac
pVV16-Mm4677-R	GTGGTGGTGAAGCTTaaccctctcgatgatc
pET28a-Rv0859-F	ATGGGTCGCGGATCCatgtccgaagaagcc
pET28a-Rv0859-R	GTGGTGGTGCTCGAGaaccctctcgatgatc
Rv0824c-RTF	TTGAACCGGTCGTCGAGAAG
Rv0824c-RTR	ACCTGGGCGACATCAGAAAG

Rv0860-RTF	GGCGAGATCGAAGACATCGT
Rv0860-RTR	AAAGTCTTCCTGCCGCTTGA
Rv1094-RTF	AGGACGTTCGAGTCCAACAC
Rv1094-RTR	AGAACGCCATGTACACCAGG
Rv3774-RTF	CAAGCTAGCGATTGTTGCCG
Rv3774-RTR	AGCGCGAGTTCTCGTAGATG
Rv0859-RTF	TTCCCGACGAGAAGCTCAAC
Rv0859-RTR	GATGCACAGCGTGATGAGTG
mIL-6-Intron-F	TCTGGCGGAGCTATTGAGAC
mIL-6-Intron-R	GATGGAAGTCTCCTGCGTGG
16sRNA-F	CCGCGGCCTATCAGCTTGTTGGT
16sRNA-R	GTAGTTGGCCGGTGCTTCTTCTCC
β-actin-zebrafish-F	ATGGATGAGGAAATCGCTGCC
β-actin-zebrafish-R	CTCCCTGATGTCTGGGTCGTC
IL6-zebrafish-F	TGCTACACTGGCTACACTCTT
IL6-zebrafish-R	CACATCCTGAACTTCGTCTCC
